# *WatFinder*: a *ProDy* tool for protein–water interactions

**DOI:** 10.1093/bioinformatics/btae516

**Published:** 2024-08-17

**Authors:** James M Krieger, Frane Doljanin, Anthony T Bogetti, Feng Zhang, Thiliban Manivarma, Ivet Bahar, Karolina Mikulska-Ruminska

**Affiliations:** Biocomputing Unit, Department of Macromolecular Structure, National Center for Biotechnology (CNB-CSIC), Calle Darwin 3, Campus UAM Cantoblanco, 28049, Madrid, Spain; Institute of Physics, Faculty of Physics, Astronomy and Informatics, Nicolaus Copernicus University in Torun, 87100, Torun, Poland; Department of Physics, Faculty of Science, University of Split, 21000, Split, Croatia; Laufer Center for Physical and Quantitative Biology, Stony Brook University, Stony Brook, NY 11794, United States; Laufer Center for Physical and Quantitative Biology, Stony Brook University, Stony Brook, NY 11794, United States; Institute of Physics, Faculty of Physics, Astronomy and Informatics, Nicolaus Copernicus University in Torun, 87100, Torun, Poland; Laufer Center for Physical and Quantitative Biology, Stony Brook University, Stony Brook, NY 11794, United States; Department of Biochemistry and Cell Biology, Renaissance School of Medicine, Stony Brook University, Stony Brook, NY 11794, United States; Institute of Physics, Faculty of Physics, Astronomy and Informatics, Nicolaus Copernicus University in Torun, 87100, Torun, Poland

## Abstract

**Summary:**

We introduce *WatFinder*, a tool designed to identify and visualize protein–water interactions (water bridges, water-mediated associations, or water channels, fluxes, and clusters) relevant to protein stability, dynamics, and function. *WatFinder* is integrated into *ProDy*, a Python API broadly used for structure-based prediction of protein dynamics. *WatFinder* provides a suite of functions for generating raw data as well as outputs from statistical analyses. The *ProDy* framework facilitates comprehensive automation and efficient analysis of the ensembles of structures resolved for a given protein or the time-evolved conformations from simulations in explicit water, as illustrated in five case studies presented in the Supplementary Material.

**Availability and implementation:**

*ProDy* is open-source and freely available under MIT License from https://github.com/ProDy/ProDy.

## 1 Introduction

The significance of accurate evaluation of protein–water interactions in the design of drugs, ligand binding, enzymatic reaction mechanisms, ion and substrate channeling, or proton/electron transfer, has long been recognized (Ladbury 1996, Verdonk *et al.* 2005, Maurer and Oostenbrink 2019). Water molecules are often active participants in the functional interactions of proteins, as they can donate or accept protons to form bridges between pairs of amino acids, form clusters, or perturb structure, thus directly impacting protein stability and function (Mattos 2002). They often facilitate binding, channeling, and transport events.

Understanding the effect of water molecules on protein structure and dynamics requires the examination of ensembles of conformations to capture recurrent patterns or the time evolution of interactions. To enable ensemble analyses, we developed a new tool *WatFinder* that takes advantage of the computing environment of the widely used *ProDy* application programming interface (API) (Bakan *et al.* 2011). *WatFinder* is designed to provide insights into key protein–water interactions that contribute to protein stability, conformational dynamics, and thereby function. Evaluation of these interactions is essential to designing small molecule modulators and assessing the effect of residue substitutions.

While comparable resources for detecting water interactions exist, they are mostly oriented toward water involvement in protein-ligand interactions and/or lack user-friendly interfaces for data processing or visualization. The currently available methods can be divided into two groups: (i) static methods, exemplified by 3D-RISM, SZMAP, or WaterFLAP (Nittinger *et al.* 2019) and (ii) dynamic methods relying on molecular dynamics (MD) simulations, such as WaterMap, GIST, WATsite, ProBiS H2O, WATCLUST (López *et al.* 2015), and AquaMMapS (Cuzzolin *et al.* 2018, Tošović *et al.* 2022). *WatFinder* is equipped with algorithms to analyze both static and dynamic objects. It offers full customization through the utilization of advanced force field parameters. It enables users to fine-tune their search criteria to achieve greater specificity and to extract the requisite level of information. Moreover, its integrated use within *ProDy* allows for advanced data processing and elaborate analyses and interpretation of the outputs in the light of the structural dynamics of protein families. Its use and utility are described in the tutorial page http://www.bahargroup.org/prody/tutorials/watfinder_tutorial/. Five case studies in the Supplementary Material illustrate its application to various systems, from membrane proteins to supramolecular machines.

## 2 Description and functionality

### 2.1 Inputs and outputs

**Figure 1.**
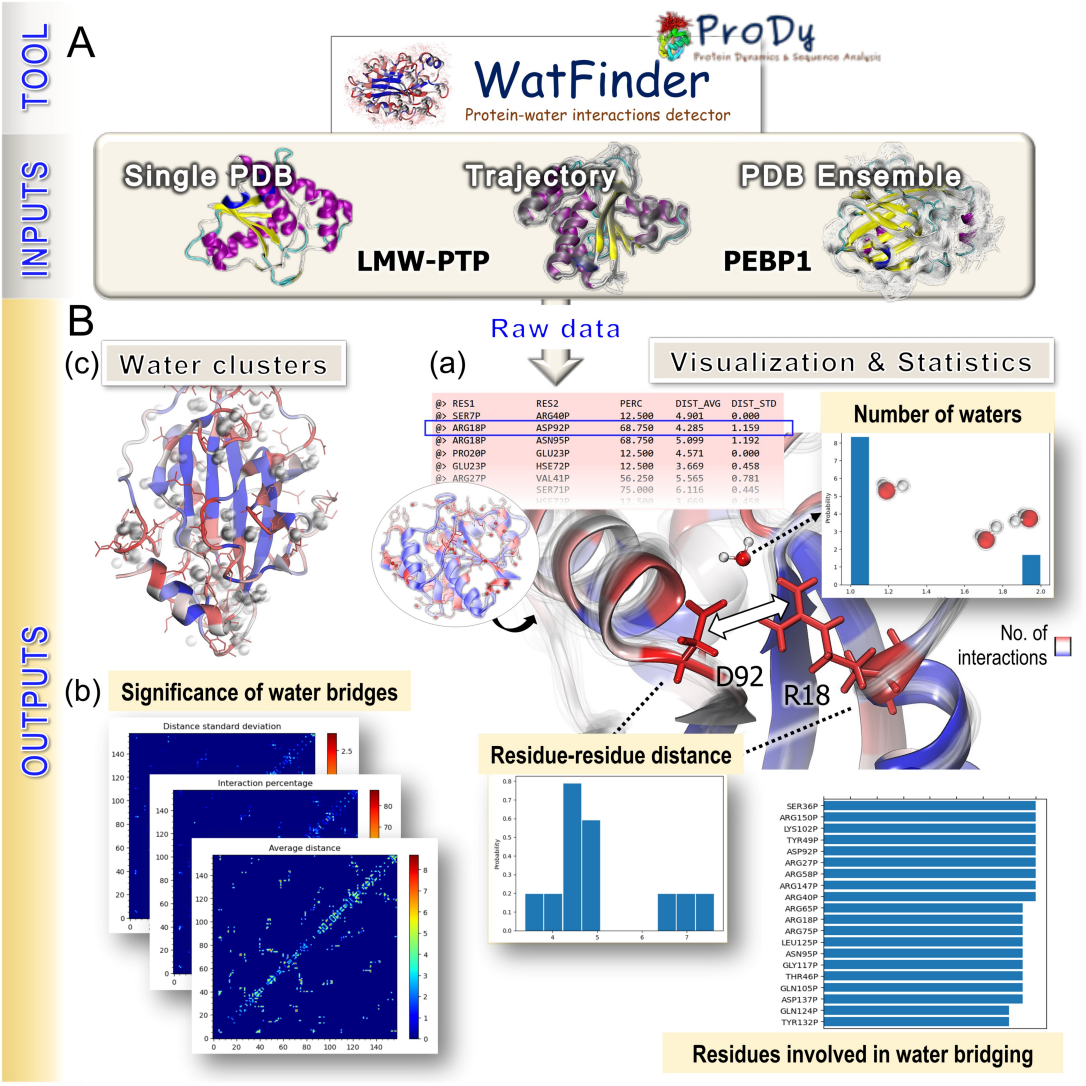
Schematic description of *WatFinder* implemented in *ProDy*. (A) *WatFinder* uses as input: (i) a single protein structure, (ii) an ensemble of structures as multiple structures resolved for the protein of interest, multiple models deposited in the PDB file, or homologous structures from multiple PDB files retrieved by *SignDy*, (iii) MD trajectory files. Here, we use for illustration the results for (i) low molecular weight protein tyrosine phosphatase [LMW-PTP; PDB: 5KQM (He *et al.* 2016)] and a short MD trajectory of the same structure using NAMD, and (ii) a multi-model ensemble of phosphatidyl ethanolamine-binding protein 1 [PEBP1, PDB: 1BEH (Banfield *et al.* 1998)]. Results for (i) are shown in the Supplementary Material. (B) Examples of (a) pairs of amino acids identified to be associated through water bridges, (b) the frequency or probabilistic occurrence of such water bridges between residue pairs, shown as heat maps based on different metrics, and (c) visualization of water clusters.

The *WatFinder* tool uses several types of input data on protein and water coordinates: A single biomolecular structure, an ensemble of structures [using the Protein Data Bank (PDB) or mmCIF format], or a series of snapshots from an MD trajectory (DCD format generated by NAMD or CHARMM) (Fig. 1A). The ensemble of structures used as input may be the multiple models deposited for a given protein (e.g. NMR models), the structures resolved for the same protein under different states (bound/unbound, active/inactive, open/closed forms, different stages of an allosteric cycle, outward- or inward-facing states, etc.), or structural homologs that may retrieved using the *SignDy* (Zhang *et al.* 2019) module of *ProDy*. The user can provide the input files or fetch the data directly from the PDB using the PDB IDs, or downstream data from *ProDy*. Other types of MD trajectory file formats, such as TRR [*GROMACS* (Lindahl *et al.* 2001)] or CRD [*AMBER* (Salomon‐Ferrer *et al.* 2013)] can be converted to multi-model PDB or to DCD format using external tools.

*WatFinder* functionalities are illustrated in Fig. 1. The outputs include: (i) raw data with a list of water bridges and associated residues; (ii) coordinates of protein structure and selected water molecules and/or water centers for visualization, and (iii) various plots and maps reflecting results from statistical analyses (Fig. 1B) using NumPy and Matplotlib.

### 2.2 Computing protein–water interactions

**Figure 2.**
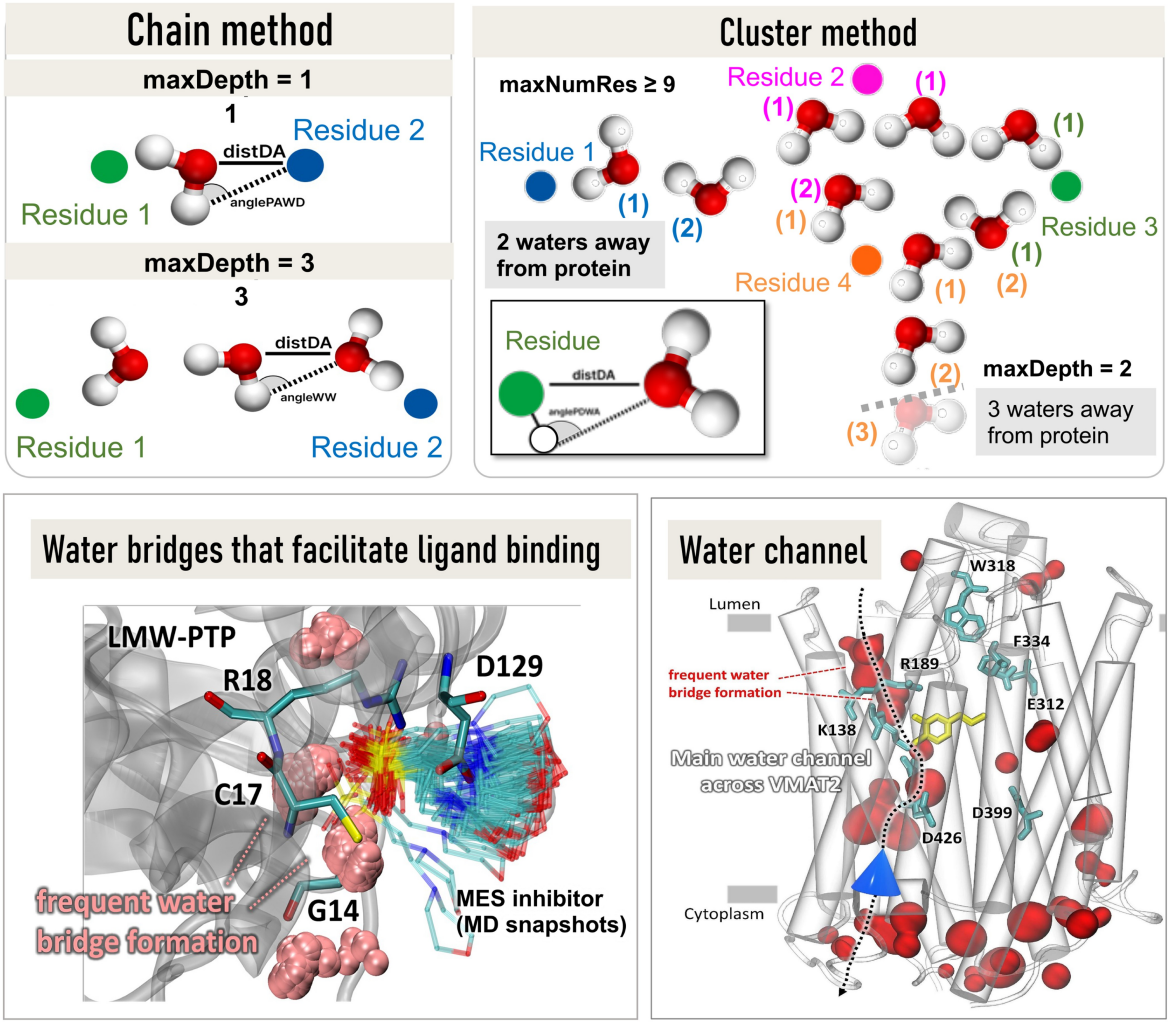
Description of two algorithms implemented in *WatFinder* for detecting protein–water interactions, and illustrative examples. *Top panels* describe the two algorithms: the chain method (*left*) and the cluster method (*right*). *Colored circles* represent hydrophilic residues interacting through water bridges. Water molecules are labeled by color-coded numbers in parentheses (on the *right*) to indicate their separations from the residues whose interactions they mediate. Distance and angle metrics are illustrated on the *left*, and lower bottom inset on the *right*. *Bottom panels*: Application of *WatFinder* to the identification of water bridges that facilitate inhibitor (MES) binding to a protein tyrosine phosphatase (*left*), and to detection of a water channel in the simulations of the vesicular monoamine transporter VMAT2. Details on these examples and others are presented in the Supplementary Material.

We have developed new classes for predicting the probabilistic occurrence of protein–water interactions, water bridges, or clusters of water molecules. *WatFinder* is adaptable and versatile, allowing users to change the geometric criteria for water bridge detection, including the threshold donor-acceptor distance (*distDA*) and orientational states (*anglePAWD, anglePDWA, angleWW*; see Fig. 2 and Supplementary Material), the threshold distance for residue-water interaction, and the number of water molecules away from residues of interest whose interactions are mediated by water bridge(s) (*maxDepth*). Water molecules can donate and accept up to two hydrogen bonds. While most bridges between residue pairs are established through one water molecule, multiple water molecules may also form a network of interactions or a cluster which is itself subject to time-dependent changes (e.g., formation of the water channel in Fig. 2, *bottom right*), thus affecting the protein’s structure and dynamics. To address such occurrences, *WatFinder* is equipped with two algorithms: (i) a *Chain method* and (ii) a *Cluster method* (Fig. 2). The *Chain method* allows the user to identify pairs of residues associated with a water bridge. The *Cluster method* additionally finds clusters involving multiple water molecules and multiple residues. Missing hydrogen atoms in the protein or water structure are added using PDBfixer/OpenMM (Eastman *et al.* 2017) or Open Babel (O'Boyle *et al.* 2011). *WatFinder* can also forecast in the absence of angular criteria for hydrogen atoms, as elaborated in the Supplementary Methods.

The major strength of *WatFinder* is its ability to analyze ensembles of structures. In addition to detecting and visualizing the water bridges occurring in each conformation, the tool identifies the most frequent bridges across the ensemble of structures, with output structures color-coded from 0 (*blue*) to 1 (*red*) (Fig. 1B). Moreover, *WatFinder* provides statistical data on the duration of interactions for each water bridge, on the average distances between hydrophilic residues involved in water bridges and their standard deviation, and on the number of water molecules participating in the interaction (Fig. 1Ba). Those data can be obtained for selected water bridges as well as the whole protein using color-coded maps (Fig. 1Bb). *WatFinder* is equipped with a cluster analyzer and characterizes the most favorable sites for water molecules within the structure [Figs 1Bc and 2*(bottom left)*].

The power of *WatFinder* as a tool for exploring protein structure and dynamics originates from its integration with the *ProDy* framework. The user can distinguish the most significant protein–water interactions and examine their impact on protein dynamics in relation to (i) the normal modes evaluated by *ProDy* using elastic network models, (ii) the principal motions extracted by principal component analysis of MD trajectories using the *ProDy* ensemble analysis module, and (iii) the signature dynamics of family members accessible via the *SignDy* module of *ProDy*.

## 3 Conclusion

The *WatFinder* module provides a fast and straightforward way to analyze protein–water interactions to obtain statistically significant outputs with user-friendly graphical and visualization tools. The tool can generate high-quality, publication-ready plots and maps. The new features provided by *WatFinder* extend *ProDy’*s capabilities to identify sites potentially affected by solvation, or prone to protonation, which may be critically important for interpreting observed behavior and designing small molecule modulators of function. From the structural and computational biology points of view, *WatFinder* offers powerful methods for complementing current studies on the impact of mutations on protein structure and dynamics, and for protein–drug/ligand design, by providing robust information on sites susceptible to interactions with water molecules, to water-mediated associations and to protonation upon exposure to water.

## Supplementary Material

btae516_Supplementary_Data

## Data Availability

Tutorials are available at http://www.bahargroup.org/prody.
