# Supplementary material for: WatFinder: a ProDy tool for protein–water interactions
Source: Bioinformatics. 2024 Aug 17;40(8):btae516. doi: 10.1093/bioinformatics/btae516 (PMC11349191; doi:10.1093/bioinformatics/btae516)
Supplement: btae516_Supplementary_Data [file btae516_supplementary_data.pdf]

SUPPLEMENTARY MATERIAL

for

WatFinder: a *ProDy* tool for protein-water interactions

James M. Krieger[1,*], Frane Doljanin[2,3,*], Anthony T. Bogetti[4], Feng Zhang[4], Thiliban Manivarma[2], Ivet Bahar[4,5,†], Karolina Mikulska-Ruminska[2,†]

[1]National Center of Biotechnology (CNB-CSIC), Madrid, Spain; [2]Institute of Physics, Faculty of Physics, Astronomy and Informatics, Nicolaus Copernicus University in Torun, Torun, Poland; [3]University of Split, Split, Croatia; [4]Laufer Center for Physical and Quantitative Biology, Stony Brook University, Stony Brook, NY, USA; and [5]Department of Biochemistry and Cell Biology, Renaissance School of Medicine, Stony Brook University, Stony Brook, NY, USA

Supplementary Results

The Supplementary Results comprise two groups of studies: First, we illustrate the utility of *WatFinder* by presenting five case studies: (1) the detection, visualization, and quantification of the formation of inter-residue water bridges as adenylate kinase transitions from the closed to open state during molecular dynamics (MD) simulations; (2) the identification of water influx and clusters into the vesicular monoamine transporter VMAT2 during MD simulations; (3) the identification of key protein-water interactions observed across an ensemble of Aurora kinase A crystal structures identified by BLAST search; (4) the detection of water interactions that facilitate ligand binding to low molecular weight protein tyrosine phosphatase; and (5) time evolution of water molecules during MD simulations of the machinery of a supramolecular assembly, the mammalian chaperonin TRiC/CCT, including timing for selected regions. Second, we provide a section on *WatFinder* default parameters selection and adjustments.

Case study 1: Identification of water bridges involved in the opening of adenylate kinase

Trajectory generation for the transition of adenylate kinase from the closed to open form. Adenylate kinase is a 214-residue enzyme that switches conformation between two distinct states: open and closed. FRET experiments indicate that the open state of apo-adenylate kinase is energetically more favorable (Scheerer et al., 2023), and so simulations initiated from the closed form should exhibit a tendency to transition to the open form. The input structure for the MD trajectory of adenylate kinase opening was prepared with the *tleap* program using the crystal structure of adenylate kinase in the closed form (PDB: 1AKE). First, the inhibitor molecule was removed, followed by solvating the protein in a truncated octahedron with a clearance of 14 Å between the protein and the edges of the octahedron. A sufficient number of sodium ions were added to neutralize the system. The protein was treated with the *ff14SB* force field (Maier et al.,

2015), the water with the TIP3P model, and the sodium ions with the Joung and Cheatham ion parameters (Joung and Cheatham III, 2008).

All simulations were run with the *pmemd* engine of the Amber20 MD package (Pearlman et al., 1995). The system was energy-minimized, then heated at 300 K for 20 ps in an NVT ensemble with weak restraints on the protein heavy atoms (1 kcal/mol) to keep the atoms at their initial positions. A timestep of 2 fs was used for integration. A Langevin thermostat with a coupling constant of 1/ps was used to maintain the temperature. Following heating, solvent equilibration was carried out in an NPT ensemble in two stages. In each stage, a Monte Carlo barostat (Allen and Tildesley, 2017) was used to maintain a target pressure of 1 atm. In the first stage of equilibration, the protein heavy atoms were kept weakly restrained (1 kcal/mol) to their initial positions and simulated for 1 ns. In the second stage, the positional heavy atom restraints were released and simulated for another 1 ns. Following equilibration, the protein was simulated in an NPT ensemble for 10 ns to capture an opening event.

The MD trajectory was prepared for analysis with *WatFinder* by first autoimaging the protein to be at the center of the water box in *cpptraj* (Roe and Cheatham III, 2013). The imaging step is important, as having the protein at the edge of the water box could introduce artifacts in the *WatFinder* analysis. Water bridges were analyzed using the *WatFinder* module implemented in the *ProDy 2.0* application programming interface (API) (Zhang et al., 2021).

Identification of water bridges implicated in the opening of adenylate kinase. To investigate which water bridges (if any) may be important in the opening mechanism of adenylate kinase, we first divided the 10 ns production trajectory of adenylate kinase opening into three portions: closed (0 to 2.5 ns), partially open (2.5 to 7.5 ns) and open (7.5 to 10 ns) (**Figure S1**). This division was based on the visualization of the trajectory and provides a basis for illustrating the ability of *WatFinder* to not only analyze trajectories but also compare selected portions of trajectories.

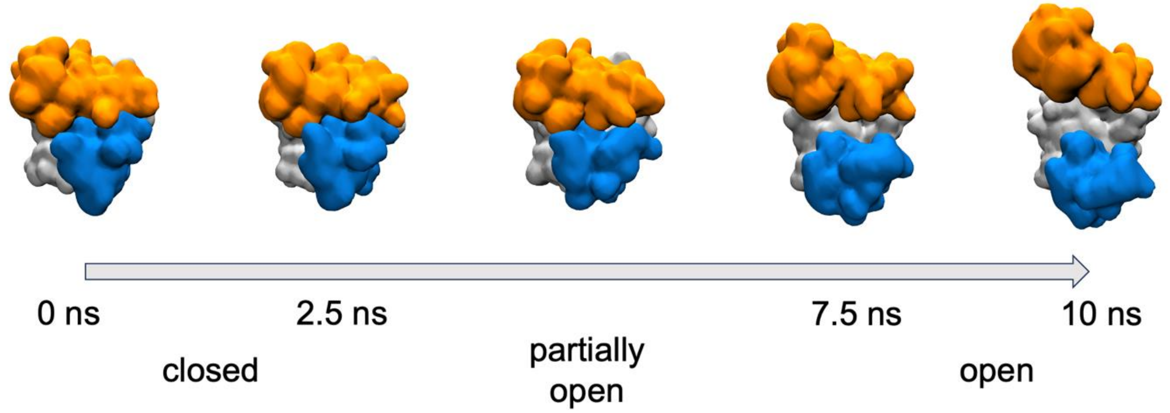

Figure S1. Evolution of adenylate kinase conformation from closed to open state during a 10 ns MD trajectory. Snapshots are shown every 2.5 ns with the lid domain in *orange,* the core domain in *gray*, and the NMP domain in *blue*. Between 0 and 2.5 ns, the protein is in the closed conformation. Between 2.5 and 7.5 ns, there is a structural transition from closed to open through many partially open conformations. Between 7.5 and 10 ns, the protein is in the open conformation.

The `calcWaterBridgesTrajectory(method='chain')`, `calcWaterBridgeStatistics()` and `calcWaterBridgeMatrix()` functions of *WatFinder* were run separately on each of the three portions of the trajectory. The resulting three matrices generated for all pairs of residues showed—for each phase—what percentage of the trajectory saw water bridge formations between each pair of residues.

After generating the inter-residue water bridge formation (percentages) matrices for each phase of adenylate kinase opening, we focused on the closed to open transition to see which water bridges were important to the opening mechanism. To this aim, we first subtracted the closed state water bridge matrix from its open state counterpart. The resulting difference matrix is shown in **Figure S2**. The entries vary from -100% to 100%, corresponding to the disruption (negative entries, color-coded from *blue* to *white*) or formation (positive entries; from *red to white*) of water bridges as the protein transitions from closed to open state.

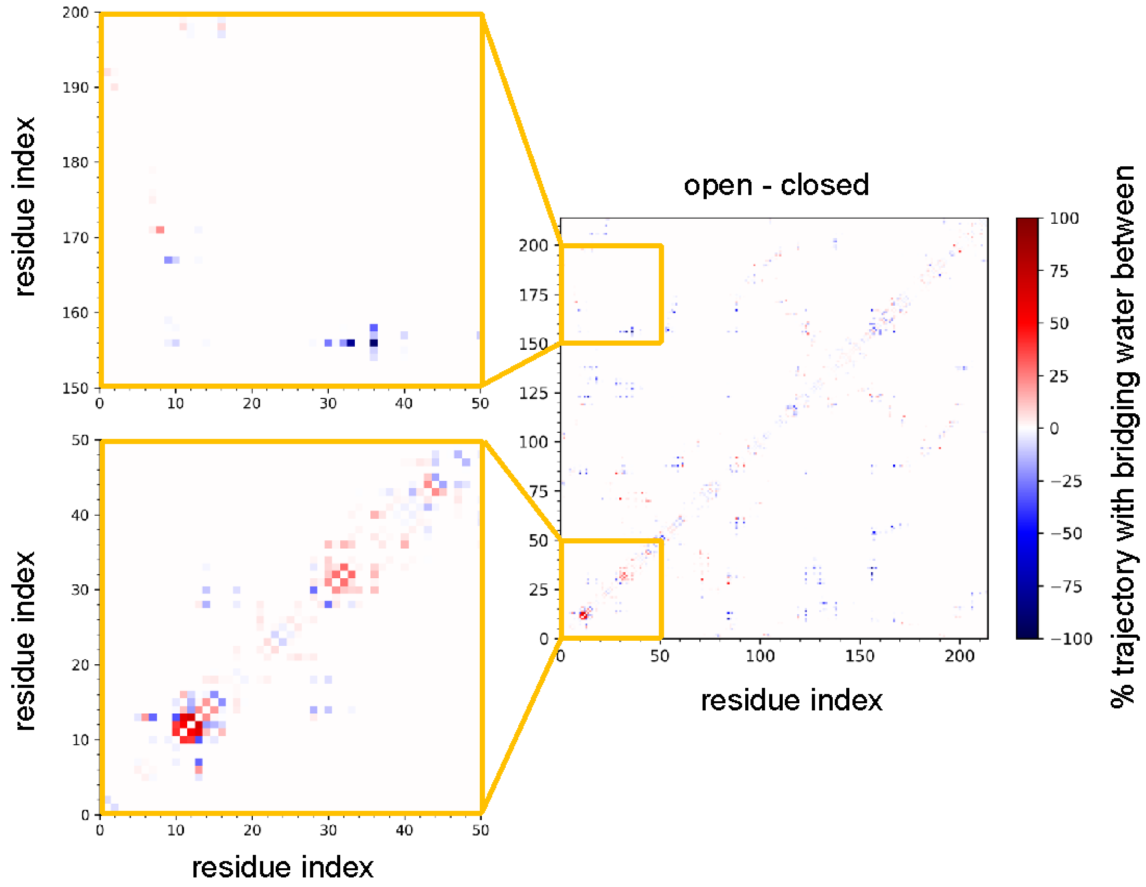

Figure S2. Inter-residue water bridge difference matrix for adenylate kinase opening. The matrix displays the distribution/frequency of water bridges formed (*red*) or broken (*blue*) between all pairs of residues, during the MD simulation of the transition from closed to open state, i.e. the entries refer to the difference {open – closed} as labeled. The color code indicates the percentage of trajectory (or the probability) of observing a water bridge involving a given residue pair when the molecule transitions from the closed to the open state. The zoomed in panels highlight the water bridges that were present in the closed state and were broken during the transition (R36 - R156 and D33 - R156; *top left*), and a network of water bridges between G12 - K13, A11 - K13 and A11 - G12 (*bottom left*) present in the open state. See **Table S1** for more details.

The residue pairs associated with the water bridges that exhibit maximal changes (either formation or disruption) between the two endpoints (closed and open) are highlighted in **Table S1**. As can be seen in the Table, the two water bridges between residue pairs D33-R156 (-80.4%) and R36-R156 (-91.2%) are lost during the opening of adenylate kinase, suggesting a role in maintaining the stability of the closed state. These residues are visualized in **Figure S3**. They are located at the interface between the lid and NMP domains, further highlighting their importance. Conversely, opening is accompanied by several new water bridges formation (between A11-G12; A11-K13; G12-K13).

Table S1. Percent change in water bridge formation (positive) or breakage (negative) between residue pairs distinguished by their significantly evolving behavior between the closed and open state portions of the trajectory.

Residue 1	Residue 2	% change
G12	K13	+65.9
A11	K13	+59.8
A11	G12	+50.2
R36	R156	-91.2
D33	R156	-80.4
G10	D84	-70.4

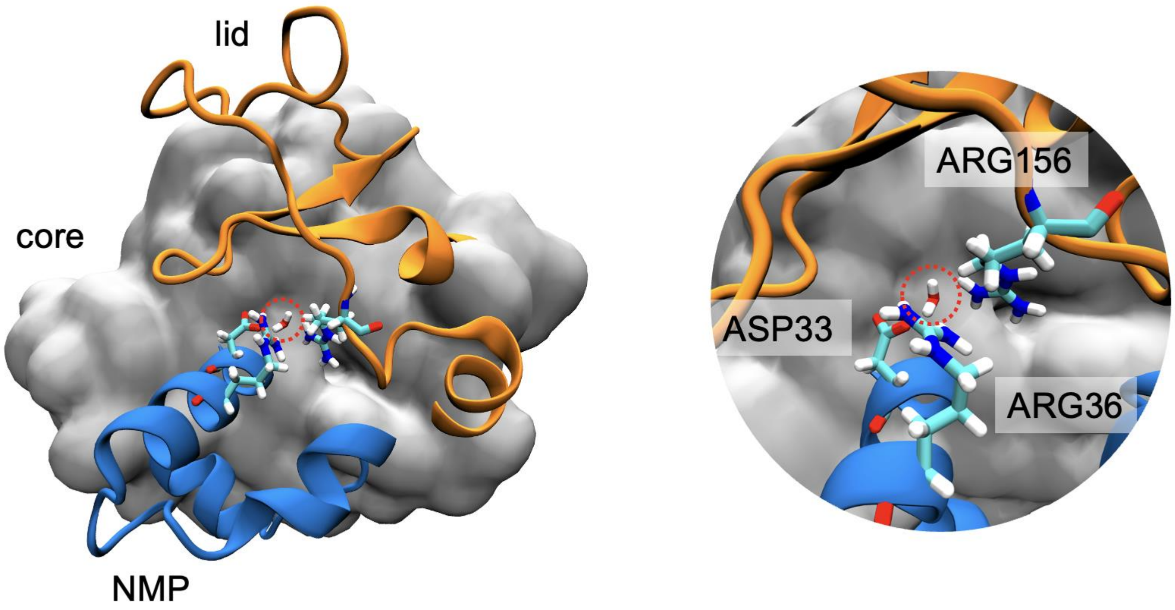

Figure S3. Visualization of the water bridges D33-R156 and R36-R156, at the interface between the lid and NMP domains of adenylate kinase.

Identification of transient water bridges formed and then broken during adenylate kinase opening. Characterization of transient states at full atomic resolution is one benefit of analyzing MD trajectories over static structures. During the simulated closed → open transition of

adenylate kinase, we identified an intermediate phase between 2.5 ns and 7.5 ns. We wondered whether any water bridges were formed solely during this phase, which would provide insight into the role of water bridges in the stabilization of transient structures during opening. To do this, we generated two additional matrices, one for the closed to partially open transition and another for the partially open to open transition (**Figure S4**).

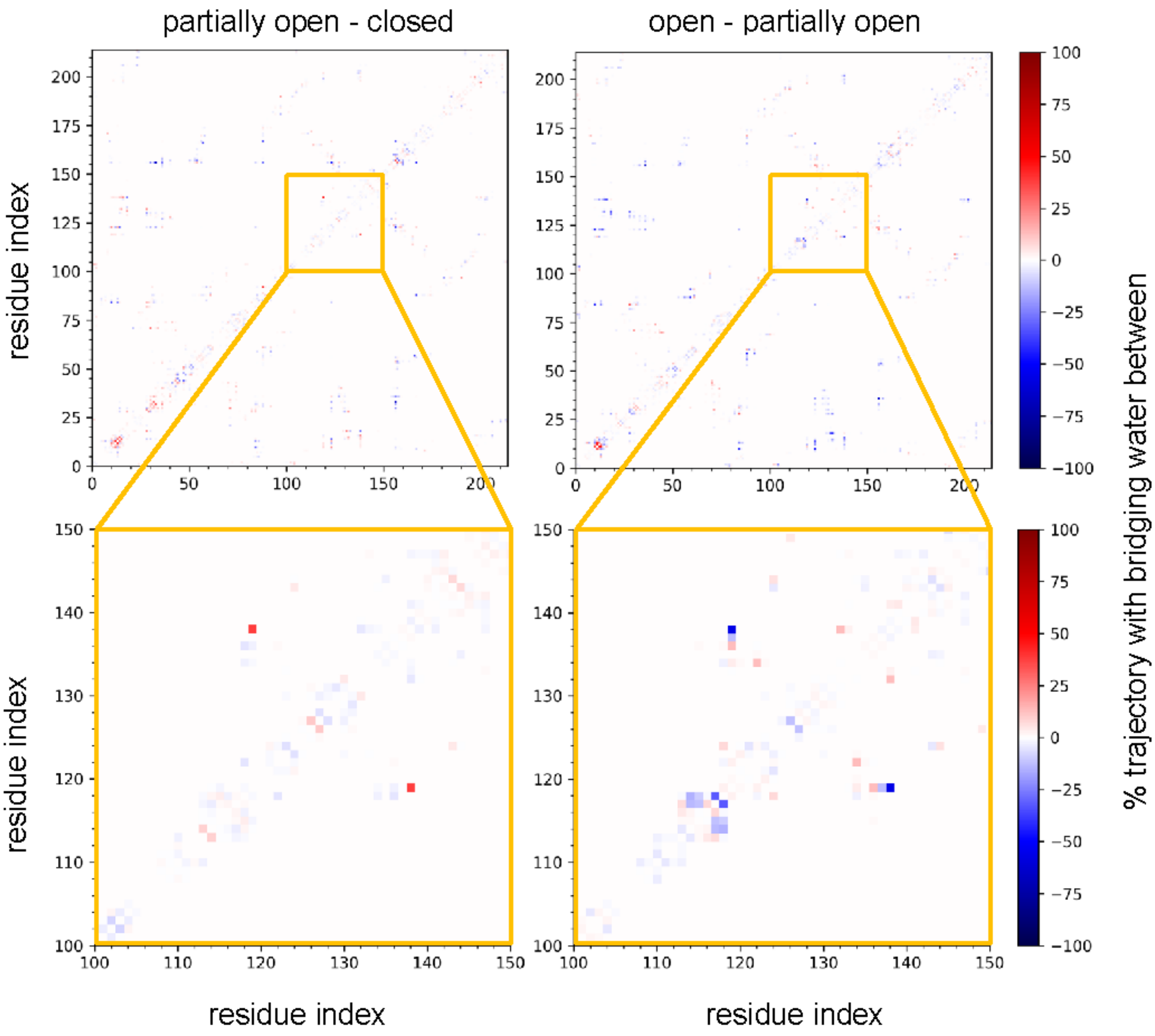

Figure S4. Inter-residue water bridge difference matrices between the two successive transitions, from closed to partially open (*top-left*) and the partially open and open (*top-right*) portions of the trajectory. The zoomed-in portions highlight the transient water bridge formed between R119-N138 during the closed to intermediate transition (*bottom-left*), which then breaks during the intermediate to open transition (*bottom-right*).

By comparing these matrices to that generated for the overall closed to open transition, the same D33-R156 and R36-R156 water bridges are observed as key to the simulated transition (**Tables S2 and S3**). However, for the closed to partially open transition, a new water bridge between R119 and N138 emerges as important (+37.8% increase from closed to partially open). This water bridge was not detected in the *full* open to closed transition because it was *lost* in the partially

open to open transition (-56.6% decrease). The water bridge between R119 and N138, therefore, forms and then breaks before the open state is achieved, suggesting its role as a transient water bridge key to the opening mechanism of adenylate kinase. This water bridge is located between two different parts of the lid domain of adenylate kinase (**Figure S5**).

Table S2. Percent change in water bridge formation (positive) or disruption (negative) during the passage from closed to partially-open state of adenylate kinase, shown for residue pairs exhibiting maximal changes

Residue 1	Residue 2	% change
R119	N138	+37.8
R88	Q92	+37.6
G12	K13	+36.4
D33	R156	-65.2
R123	R156	-48.8
K57	K166	-45.2

Table S3. The percent change in water bridge formation (positive) or disruption (negative) during the passage from partially-open to open state of adenylate kinase, shown for residue pairs exhibiting maximal changes

Residue 1	Residue 2	% change
A11	K13	+46.8
A11	G12	+40.6
G10	A11	+31.0
R36	R156	-63.8
R119	N138	-56.6
V59	R88	-44.4

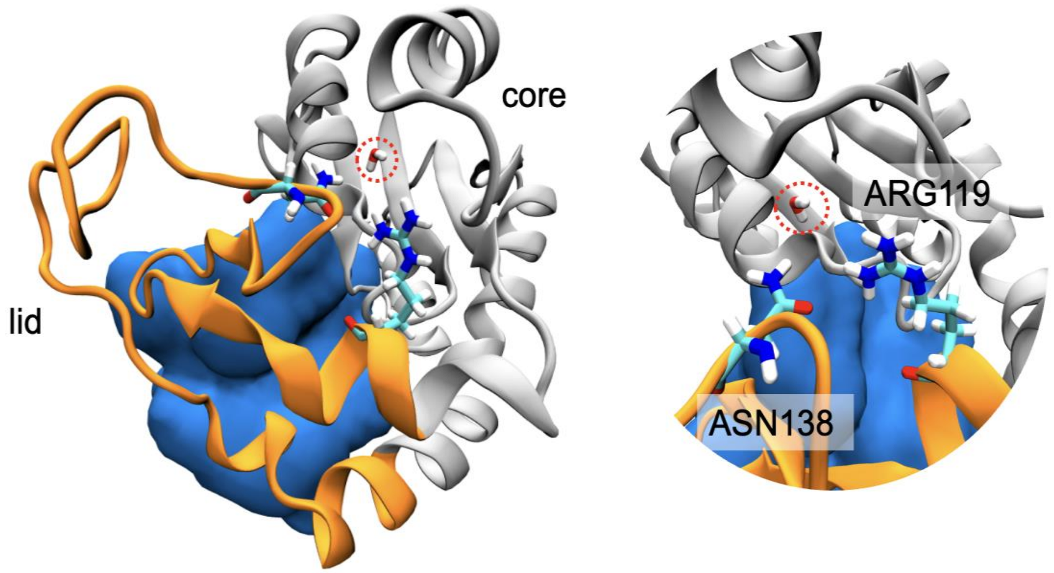

Figure S5. Visualization of a key intermediate water bridge R119-N138 which is located between two distal regions of the lid domain.

Discussion. This case study illustrates the application of *WatFinder* to a short MD trajectory performed for a small protein (214 residues) in explicit solvent. *WatFinder* analysis of short MD trajectories like this one is feasible on a single CPU core and only takes a few minutes.

Conclusion. We presented a brief investigation of water bridges formed or broken during the transition of adenylate kinase from closed to open, automated by *WatFinder*. The tool permitted us to clearly identify water bridges between the lid and NMP domains that are important to the opening mechanism, and also a transient water bridge between the lid and core domains of adenylate kinase. The identification of transient water bridges highlights the utility of analyzing MD trajectories with *WatFinder* compared to just analyzing static structures, as such intermediates could serve as targets for modulating the transitions.

Case study 2: Vesicular monoamine transporter 2

Vesicular monoamine transporter 2 (VMAT2) is a proton-dependent transmembrane antiporter composed of 476 residues, responsible for loading monoamine neurotransmitters into synaptic vesicles (Dalton et al., 2024). In this example, *WatFinder* enabled us to analyze the all-atom MD simulations of VMAT2 in a membrane to detect the flux of water across the antiporter structure. The simulation details are available in a recent publication (Dalton et al., 2024). The analysis of *WatFinder* is based on 210 frames, which were superimposed (aligned) using the VMD program (Humphrey et al., 1996).

Results. Identification of highly stable water bridges formed within the transporter VMAT2. To investigate where water bridges were formed in the VMAT2 structure, we first used the `calcWaterBridgesTrajectory()` function with `method='cluster'`. The cluster algorithm provides information about water clusters and between more than two residues from the protein, making it ideal for the analysis of water channels across a transmembrane protein like VMAT2. Different parameters of the cluster method algorithm can be adjusted to personalize the analysis of a variety of systems. For instance, water molecules may remain stably bound for extended durations to well-defined locations in some systems, but in others, they may be diffusing or channeled through an opening. Parameters such as `maxDepth` (**Figure 2**) or `maxNumRes` (maximum number of water molecules plus protein residues in a cluster) are crucial to adjust to make sure that the method of analysis matches the type of process being examined. In the present case (and in the absence of prior knowledge of the expected behavior), we adopt the default parameters, which should be general to most systems.

Next, the `savePDBWaterBridgesTrajectory()` function was used to save the conformations (in PDB format) that contain the detected water bridges; these conformations were further analyzed with the `findClusterCenters()` function. Additionally, parameters such as `distC` (maximum distance between independent water molecules in the cluster and counted between oxygen atoms in Å) and `numC` (minimum number of water molecules in a cluster) were readjusted to find the most suitable parameters for the system. First, more relaxed parameter values were tested, where the distance between water molecules (`distC`) was set

to *0.5* Å, and the minimum number of waters in a cluster (numC) was set to 4. This approach helped identify two possible water channels across the VMAT2 structure (along the *black, dotted arrows* in **Figure S6**) and a large number of possible water-protein interactions in the cytoplasmic and lumenal regions. The identified channels correspond well to what was observed independently in a previous study ((Dalton et al., 2024), *see Figure 5 in the manuscript*). In **Figure S6,** the orientation of the transporter is similar to that reported earlier, and key residues are displayed to ease the comparison.

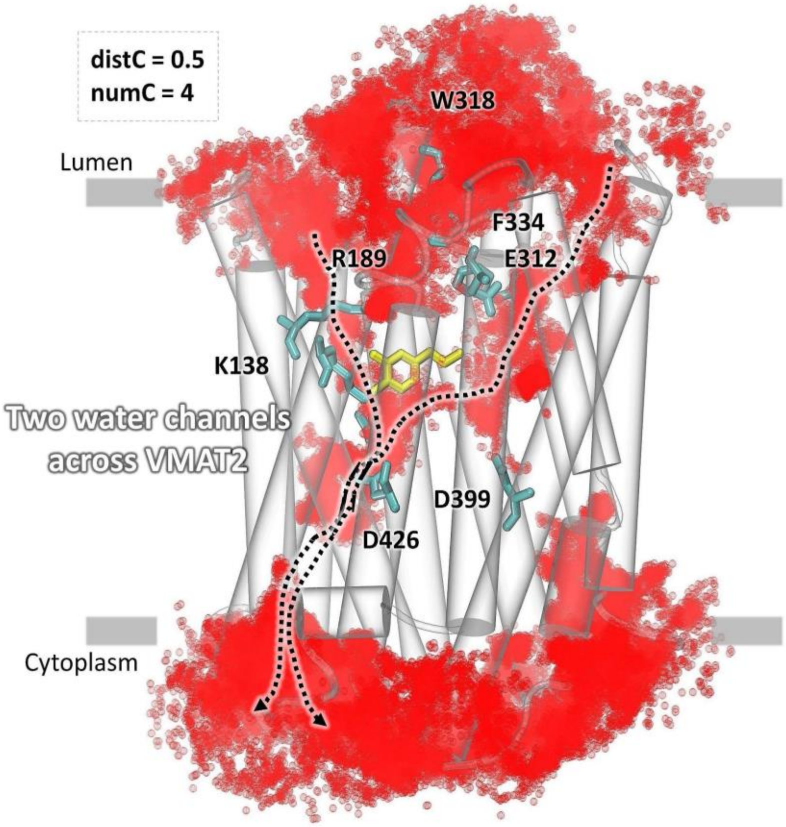

Figure S6. Water clusters and channeling pathways identified for VMAT2. The MD trajectories generated for VMAT2 were analyzed using the `findClusterCenters()` function with parameters `distC=0.5` and `numC=4`.

The analysis was repeated with more restrictive parameters to obtain only the most significant water interactions with the protein. The adoption of the parameters `distC=0.2` and `numC=5` significantly decreased the number of detected clusters (**Figure S7**, also shown in **Figure 2**), revealing one main water channel across the VMAT2 (denoted by the *black, dotted arrow*). The use of other parameters, such as `distC=0.3` and `numC=10`, did not change the outcome, revealing that the main flux of water is robustly maintained (**Figure S8**).

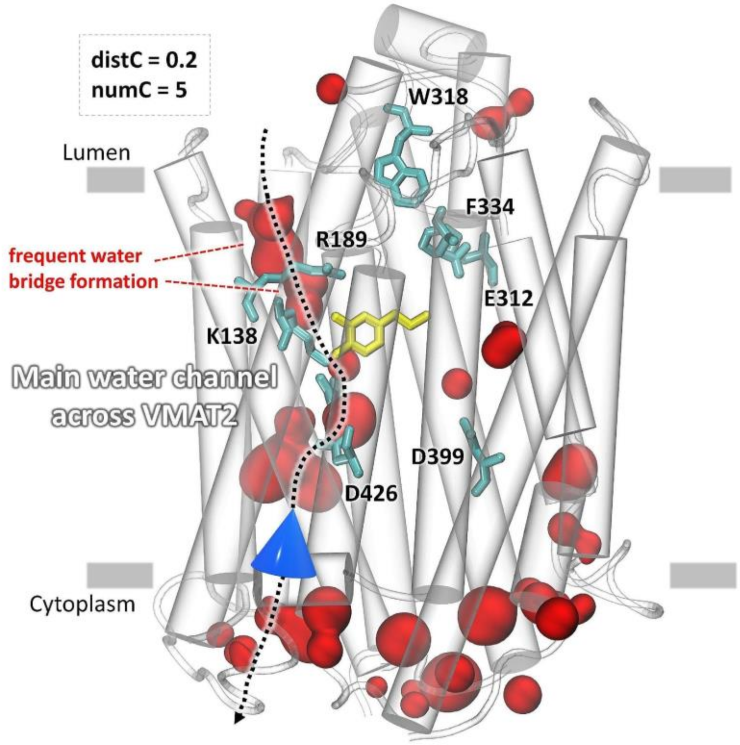

Figure S7. Water clusters for VMAT2 were identified using the `findClusterCenters()` function with parameters `distC=0.2` and `numC=5`. The blue funnel indicates the place where the free flow of water occurred, as a result of which no specific interactions with the protein were observed.

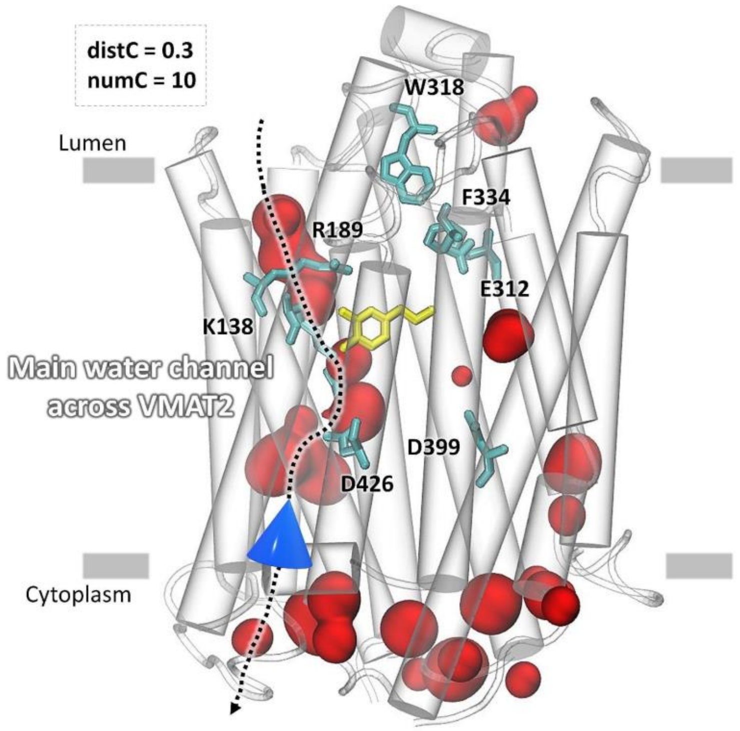

Figure S8. Water clusters for VMAT2 were identified using the `findClusterCenters()` function with parameters `distC=0.3` and `numC=10`.

Discussion. This case study shows the application of *WatFinder* to a short MD trajectory of a medium-sized transmembrane protein (~500 residues) in a phospholipid bilayer. The *WatFinder* analysis of the MD trajectory (210 frames) like this one on six CPU cores takes less than 2 minutes (for the `'chain'` method) or around 2.2 minutes for the `'cluster'` method. Identification of the water clusters using `findClusterCenters()` takes around 2 minutes with default values. The time of calculation will decrease with a bigger `distC` parameter. To optimize the results, we tested various values of `distC` and `numC` to obtain a sufficient number of clusters that illustrate water influx through the protein, while keeping `distC` as small as possible and `numC` as large as possible (see **Supplementary Table 2**).

Conclusion. The application to the VMAT2 MD trajectory demonstrates another utility of *WatFinder*, this time as a detector of water flux across a transmembrane protein. The advantage of *WatFinder* is not only the detection of water flux within a protein structure but also the detection of functionally relevant interactions between the membrane protein and water molecules.

Case study 3: Aurora kinase A

Aurora kinase A (AurA) is a mitotic serine/threonine kinase composed of approximately 300 residues. It plays various roles, including the regulation of cell cycle progression (Marumoto et al., 2002) and the phosphorylation of numerous proteins such as BRCA1, PLK1 (Macůrek et al., 2008), p53 (Katayama et al., 2004), and TPX2 (Bird and Hyman, 2008). It has been shown (Cyphers et al., 2017) that the catalytic activity of AurA is controlled by conformational changes at its conserved DFG motif. The spindle-associated protein Tpx2 activates AurA by stabilizing a water-mediated allosteric network that links the C-helix to the active site through an unusual polar residue in the regulatory spine. The polar spine residue and water network of AurA (shown as W1 and W2 in earlier work (Cyphers et al., 2017)) and identified here by *WatFinder* (**Fig. S9**)) are essential to the phosphorylation-driven activation. This kind of water network has been observed in other related kinases as well, suggesting that variations in the water-mediated hydrogen bond network may play a role in the regulatory diversification of protein kinases in general (Cyphers et al., 2017). The authors proposed that Q185 and the associated water network represent a polar analog of the regulatory spines of other protein kinases, performing a related function.

Method. In this example, we combined *WatFinder* with an already available *ProDy* functionality - a BLAST search - to find persistent/conserved protein-water interactions across various AurA structures deposited in the Protein Data Bank. This kind of approach driven by structural data for an ensemble of homologs can be applied to any other system to identify significant water bridges that are shared across multiple PDB structures, including those belonging to various species. The ensemble size and content will depend on the sequence identity applied in the BLAST search. The BLAST search is described in the Ensemble Analysis tutorial of *ProDy*. We additionally provide a Jupyter Notebook file in the *WatFinder* tutorial to explain each step of the analysis.

Identification of highly stable water bridges in AurA-independent structures. To identify regions in AurA structures where the water bridges are most frequently formed, we first used the sequence in the corresponding PDB structure (PDB: 1OL5) to screen the PDB database through a BLAST search (using the `blastPDB()` function available in *ProDy*), as detailed in the Ensemble Analysis tutorial of *ProDy*.

To narrow down the number of retrieved PDB structures to those that are highly similar to AurA, we applied a sequence identity (`seqid`) parameter of 90%. This resulted in 192 structures. These structures were saved based on the chain indicated by BLAST and further checked to ensure that they contained water molecules, using the `filterStructuresWithoutWater()` function. This led to 156 structures containing water molecules, each sharing at least 90% sequence identity with respect to the original AurA structure with PDB code 1OL5. Here, we focused on close homologs, but the analyses can be readily adopted to more distant homologs by changing the sequence identity threshold.

Once we selected the PDB structures for analysis, we added missing hydrogens to multiple PDB structures, where needed, using the `fixStructuresMissingAtoms()` function, and we aligned the resulting structures using the `matchChains()` function. After those preparations, the structures were ready for *WatFinder* predictions. We applied `calcWaterBridges()` and `savePDBWaterBridges()` using default parameters to identify and save water bridges for each of the 156 structures. Saved structures with water bridges can be independently uploaded to any graphical visualization program. They contain the `'wb_align__'` prefix.

Next, we used the `findClusterCenters()` function with `selection='resname HOH and name O'`, `distC=0.2`, and `numC=5` to identify water clusters that are shared among AurA structures. See the previous example for the description of these parameters. The water clusters obtained by this approach are displayed in **Fig. S9**. Among them, we can find two, W1 and W2, discovered earlier (Cyphers et al., 2017) in the context of water-mediated hydrogen bond networks critical to AurA kinase activity.

Discussion. This case study shows the application of *WatFinder* to various crystal structures of a small protein (~265 residues). The *WatFinder* ensemble analysis (of 156 protein structures) takes around 1 minute using a single CPU. Additionally, identifying water clusters using the `findClusterCenters()` function takes less than 1 minute, even with large `distC` values. This efficiency is due to the significantly relatively small numbers of waters in crystal structures compared to MD trajectories. The values of `distC` and `numC` were adjusted based on the number and diversity of analyzed structures. We recommend increasing the value of `distC` if the side chains exhibit greater flexibility in the analyzed crystal structures.

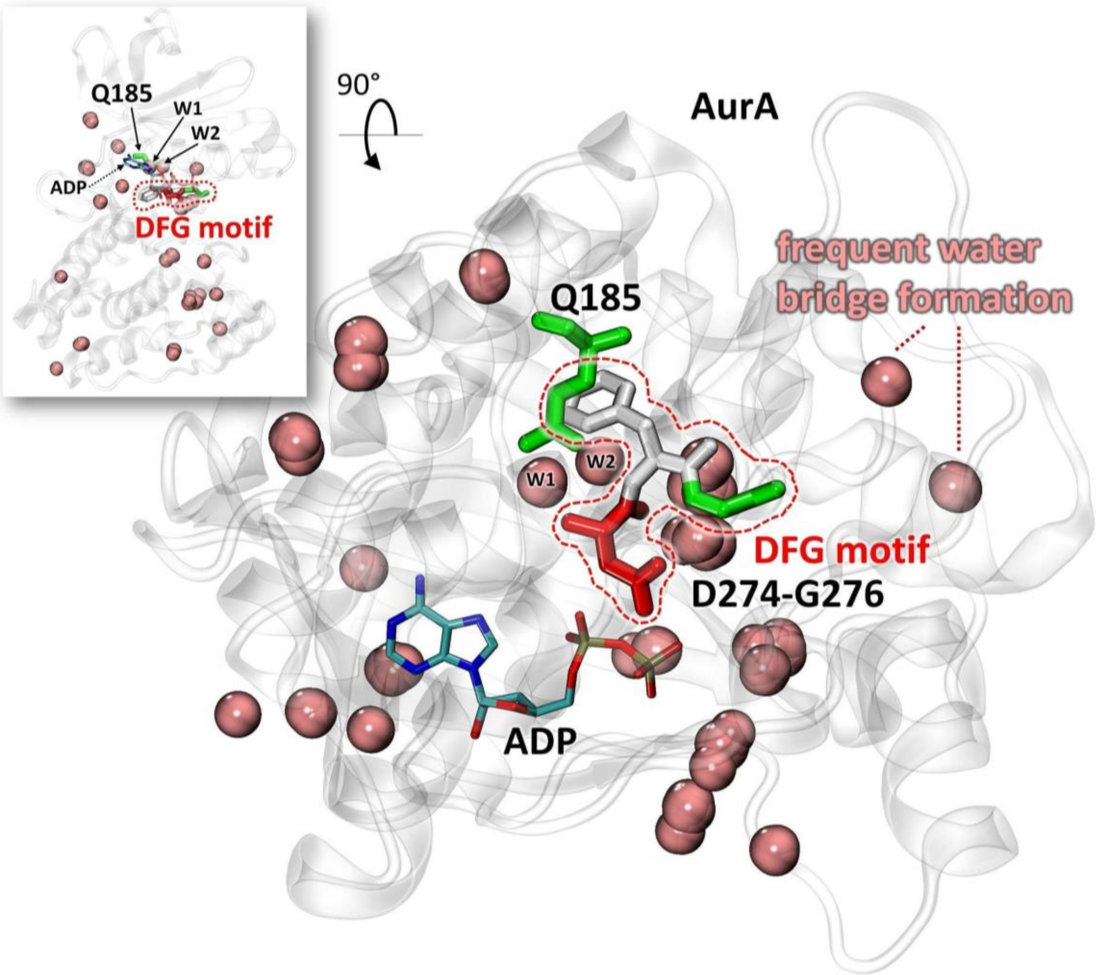

Figure S9. Water clusters identified for Aurora kinase A and its homologs sharing 90% sequence identity. Crystal structure of AurA (PDB code: 1OL5) is displayed as a transparent *ribbon diagram*. The clusters of water were predicted based on 156 crystal structures that contained water molecules. We used `distC=0.2` and `numC=5` parameters to obtain these results. Clusters W1 and W2 correspond to the water clusters identified in previous work (in *Figure 1* of Cyphers et al., 2017). DGF motif residues D274-F275-G276 and their spatial neighbor Q185, displayed in *bold sticks*, are part of the water-mediated hydrogen bond network.

Conclusion. We demonstrated another utility of *WatFinder*, this time combined with BLAST search to retrieve sequence homologs that have been structurally resolved. We analyzed the retrieved ensemble of structures (based on a user-defined sequence identity threshold) to identify water bridges that frequently occur among the multiple structures for the query protein resolved by different experimental groups) or homogenous (other species) structures. This approach can be applied to any other system to identify critical water-interaction regions as well as places in which water molecules should be included in molecular dynamics simulations to avoid instability of the protein structure.

Case study 4: Low Molecular Weight Protein Tyrosine Phosphatase

The low molecular weight protein tyrosine phosphatase (LMW-PTP) is a promising target for cancer and diabetes/obesity treatments, as a regulator of many signaling pathways. Therefore, there is therapeutic interest in developing inhibitors to modulate LMW-PTP activity, as discussed previously (He et al., 2016). He et al. also provided a crystal structure of LMW-PTP with MES inhibitor (PDB: 5KQM), which we used in this study to illustrate the potential utility of *WatFinder* in assisting drug design. LMW-PTP is composed of 158 residues.

Method. In this example, we performed 50 ns all-atom MD simulations using NAMD (Phillips et al., 2005) with the CHARMM27 force field to obtain the dynamics of: (i) LMW-PTP in the presence of the inhibitor MES and (ii) LMW-PTP alone. The inhibitor's energy parameters were obtained using SwissParam (Zoete et al., 2011). We adopted the following protocol: 0.2 ns of water equilibration and 10,000 steps of minimization, next 0.35 ns of heating from 0 to 300 K, and 0.15 ns of equilibration of the whole system before initiating the productive MD run. We used explicit water (TIP3P) and 0.15 mol/L NaCl concentration to neutralize the system. The *WatFinder* analysis was based on 500 frames (50 ns for each system), which were structurally aligned using VMD (Humphrey et al., 1996).

Results. To investigate the most significant LMW-PTP - water interactions, we used the `calcWaterBridgesTrajectory()` function with the `'chain'` method. We followed the protocol used for Case Study 2, i.e., `savePDBWaterBridgesTrajectory()` to save the conformations in PDB format that contained the detected water bridges. These were analyzed with the `findClusterCenters()` function. We used `distC=0.2` and `numC=10` to select the water interactions that are concentrated at precisely defined sites. This approach helped us identify the most significant protein-water interaction sites, displayed as *dotted green spheres* in **Figure S10**. The greater the intensity and size of the water cluster, the more conserved the site we identified. The results imply that the inhibitor (MES) binding site (see initial pose in **Figure S10a** and MD snapshots in **Figure S10b**) is predisposed to water interactions which assist in binding, or stabilizing the bound, inhibitor. Further analysis of the LMW-PTP MD generated in the presence of MES helped detect water clusters in the close vicinity of the inhibitor binding site. The water clusters were observed to support the interaction of selected inhibitor-binding residues with MES, as shown in **Figure S11**).

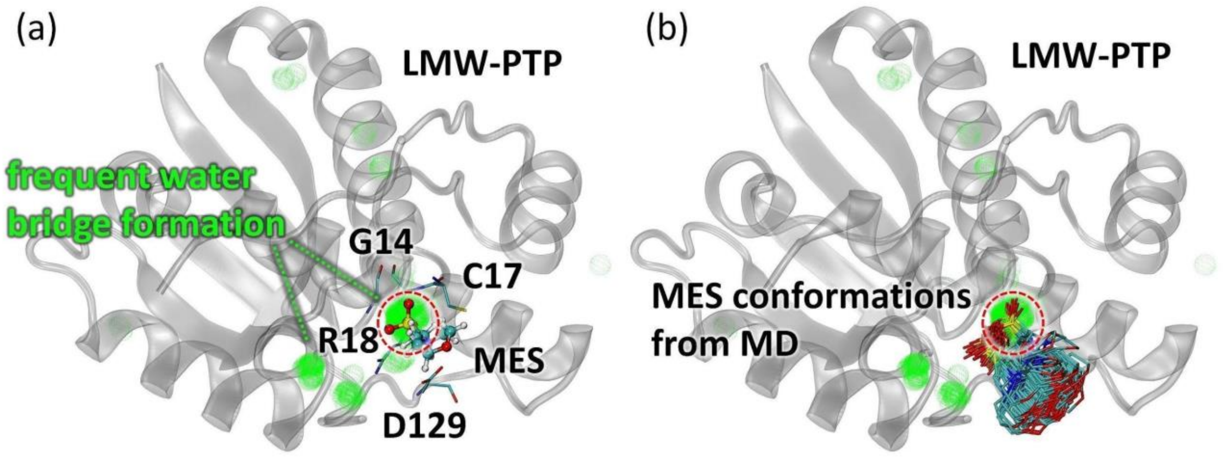

Figure S10. Water clusters identified for LMW-PTP structure in all-atom MD simulations of LMW-PTP. The water clusters, shown as *green dotted shapes*, were identified based on a 50 ns MD run (represented by 500 frames) in the absence of inhibitor (panel **a**). The inhibitor MES (enclosed, *springs/balls*) is displayed to show its original location (in panel a). MD simulation of LMW-PTP with MES inhibitor (panel **b**) showed the high stability of the inhibitor, coordinated by residues that form water bridges, mainly G14, C17, R18, and D129. **See also Figure S11.**

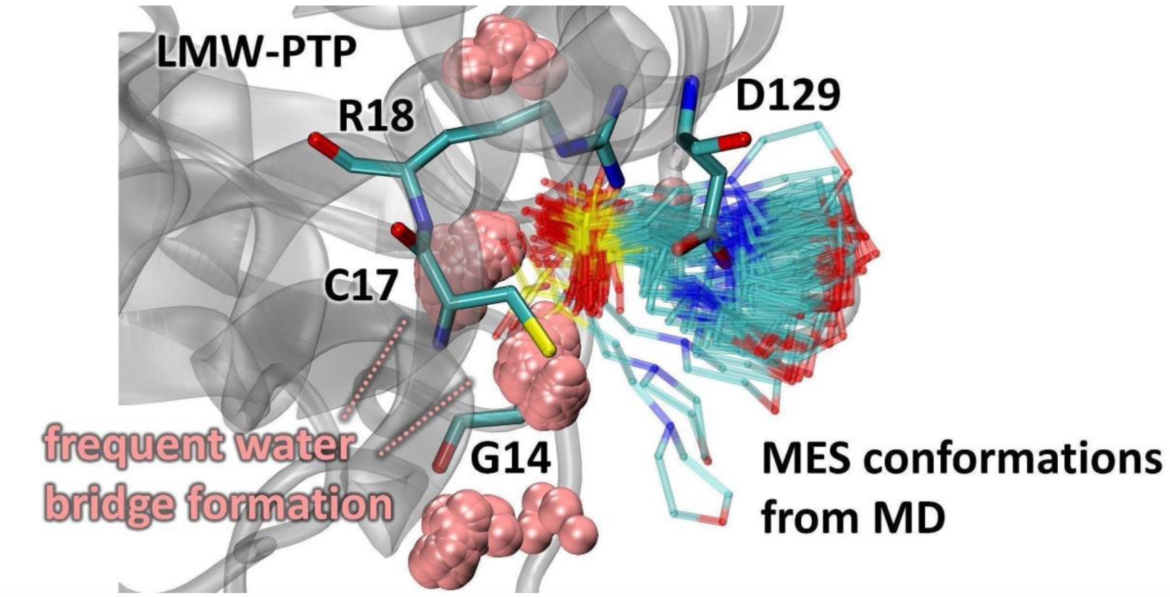

Figure S11. Water clusters identified for LMW-PTP in an all-atom MD simulation in the presence of the inhibitor MES. Water clusters obtained with *WatFinder* analysis are displayed as pink spheres for better visualization. MES conformations sampled along the trajectory are displayed.

Discussion. This case study shows the application of *WatFinder* to a short MD trajectory (50 ns) of a small protein in an explicit solvent. The current *WatFinder* analysis of the MD trajectory (500 frames) takes less than 3 minutes with default parameters for hydrogen bonds. We used

`distC=0.2` and `numC=10` to obtain precisely defined water clusters, which were observed at least ten times (or for a total duration of 1 ns).

Conclusion. *WatFinder* predicts in the present case study a number of key protein-water interactions that contribute to stabilizing the bound inhibitor. The analysis also points to potential druggability spots with good solubility, which may serve as target sites for ligand binding. *WatFinder* therefore might serve as an auxiliary tool for supporting drug discovery and development.

Case study 5: The mammalian chaperonin TriC/CCT

The chaperonin containing tailless complex polypeptide 1 (CCT) or tailless complex polypeptide 1 ring complex (TRiC) is a large molecular machine formed of two identical back-to-back, hetero-octameric (8-subunit) rings that enclose misfolded or unfolded substrate proteins to promote accurate folding in an ATP-dependent manner. A number of cryo-EM studies have revealed the conformational cycle of this chaperonin (Kim et al., 2024), capturing open conformations in the apo (nucleotide partially preloaded) and ATP-bound states that engage unfolded substrate proteins, a closed conformation following ATP hydrolysis that promotes folding, and highly dynamic open conformations again in the ADP-bound state following phosphate release that releases folded protein products. We focused on MD simulations of the ADP-bound open state to illustrate the capability of *WatFinder* to analyze large systems.

Methods. Along with our previous study of the dynamics of CCT in these various states (Zhang et al., 2021), we also ran some all-atom MD simulations. Our starting structures were from an early cryo-EM study where the subunit identity could not be unambiguously assigned (Cong et al., 2012), so all the subunits were treated as CCT2. We used the ADP-bound structure (PDB code: 4A0V), modeled the missing atoms with Maestro (Schrödinger package), and removed the ADP. The structure was embedded in a box of TIP3P water with 10 Å padding from the protein and 0.15 mol/l sodium chloride, yielding a full simulation system of 707,044 atoms. All-atom MD simulations (20-25 ns) were performed using NAMD (Phillips et al., 2005) and the CHARMM27 force field (Vanommeslaeghe and MacKerell Jr, 2015). We adopted the following protocol: 0.2 ns of water equilibration and 10,000 steps of minimization, next 0.35 ns of heating from 0 to 300 K, and 0.15 ns equilibration of the whole system before initiating the production of the main MD run. The analysis of *WatFinder* was based on 30 frames which were first superimposed.

WatFinder analysis used five different selections of substructures with increasing size to illustrate the time scalability of the method. Corresponding regions of the protein complex were selected together with the keyword option `expand_selection=True` to include all residues within the bounding boxes spanned by the maximum and minimum x, y and z coordinates of the protein selection. A final selection was created in each case that included all atoms from all the expanded selections across the full set of frames.

A large-scale analysis of selections expanded around 1 subunit (selection 1), 4 subunits (selection 3), a full 8-subunit ring (selection 4), and the full 2-ring system of 16 subunits (selection 5; see

Figure S12) was performed using Python 3.8.15 on a 64-core (hyper-threaded) node with a 2.6 GHz Xeon Gold 6142 CPU and 754 GB RAM on an Asus Z11PG-D24 cluster. Multiple analyses were conducted simultaneously using `calcWaterBridgesTrajectory()`, each launching 30 processes at once to analyze the first 30 frames in parallel. Calculations were repeated 10 times for each selection to analyze the dependency of the computing/analysis time on the selection size. A linear regression was calculated with scikit-learn 1.5.0 using all the calculated times and the number of atoms in thousands.

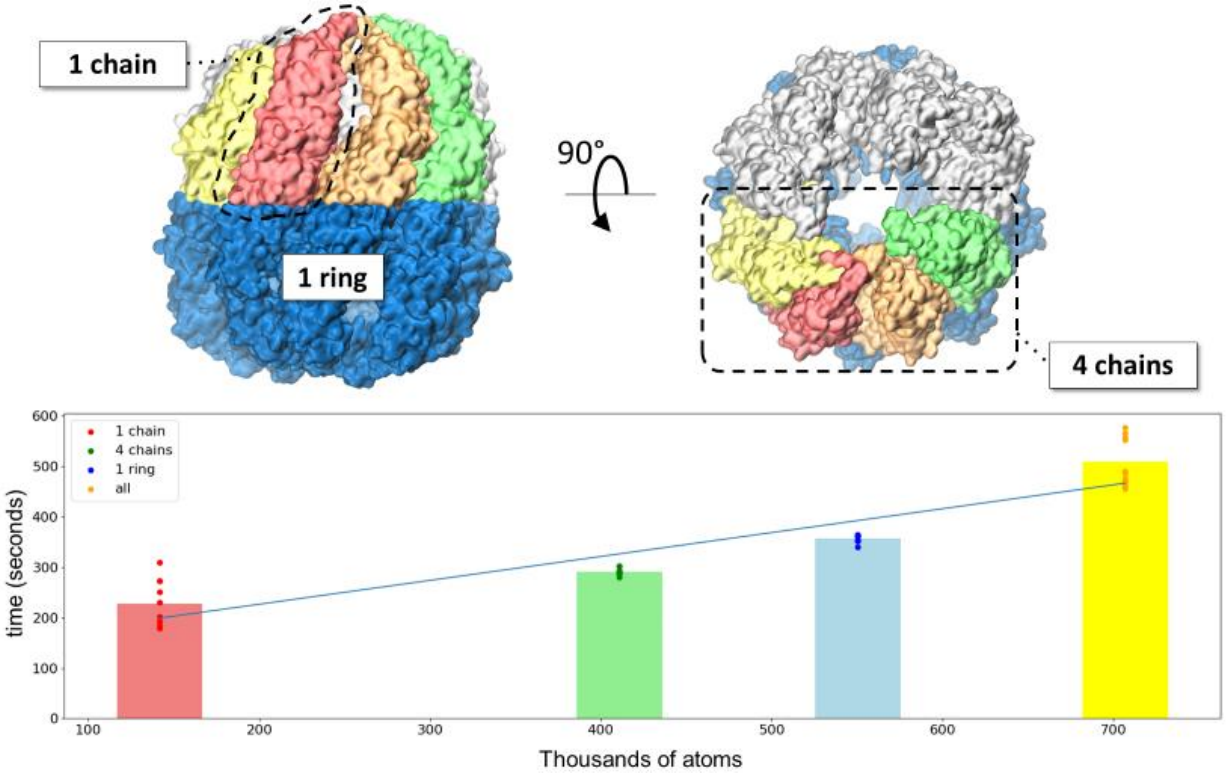

Figure S12. Time taken to analyze water bridges in different substructures of the CCT complex defined by various selections shown on the cryo-EM structure. The time it takes in seconds to analyze water clusters using `calcWaterBridgesTrajectory()` is plotted against the total number of atoms in the analyzed system. The dots show the results from 10 repeated calculations for 30 frames and the bars show the average values. The line comes from linear regression of the number of thousands of atoms against the time in seconds and has an equation of $y = 0.47\,x + 132$, indicating a 132 second portion independent of system size.

The full pipeline was run on a 224-core (hyper-threaded) cluster head node with 2.95 MB RAM using a Jupyter notebook and repeated using a script. It includes analysis of expanded selections based on 1 (selection 1), 2 (selection 2) and 4 subunits (selection 3) that used the first 10 frames. The first two steps using the functions `calcWaterBridgesTrajectory()` and `savePDBWaterBridgesTrajectory()` were ultimately run with multiple frames in parallel. Finally, the `findClusterCenters()` step was run with different parameter choices in the notebook and just once for the script. The results from the script are shown in **Figures S13**.

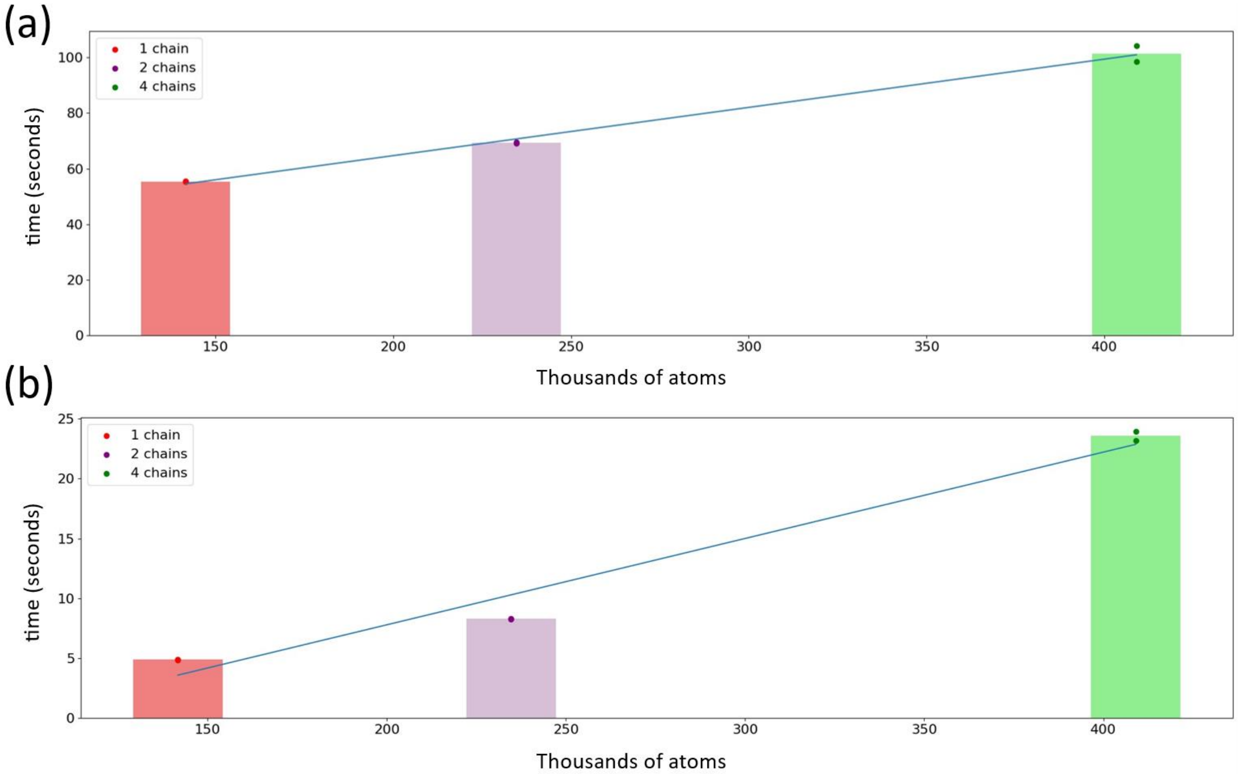

Figure S13. Time cost of analyzing water bridges in regions of the CCT complex defined by various smaller selections. (a-b) The time taken in seconds to analyze water clusters using `calcWaterBridgesTrajectory` **(a)** and the time taken in seconds to save the output for the next analysis step using `savePDBWaterBridgesTrajectory` **(b)** are plotted against the total number of atoms in the analyzed systems. The dots show the results from two repeated calculations with 10 frames and the bars show the average values. The line comes from linear regression of the number of thousands of atoms against the time in seconds for both steps with equations of $y = 0.17\,x + 30$ and $y = 0.07\,x - 6.6$, respectively.

Subsequently a detailed analysis was run for the selection based on a single subunit (selection 1; including portions of its neighbors within the same ring and between rings). This analysis focused on frame 22 to frame 31, which were selected based on analysis of the root-mean-square deviations (RMSDs) between frames as described in the **Results** section (see **Figure S14**). The `findClusterCenters()` step was again run with several different parameter choices and the combination `distC=0.8, numC=2` was found to be the best.

Results. The CCT chaperonin complex has 16 subunits organized as two back-to-back 8-subunit rings (see **Figure S12**, *top*). Each constituent subunit or chain has roughly the same structure and behavior with an equatorial domain that binds and hydrolyses ATP and forms the inter-ring interface, an apical domain that forms the lid that closes around the substrate protein, and an intermediate domain between them that couples their dynamics and provides substrate interaction sites (Kim, et al., 2024). An MD simulation of this system was analyzed both in a general way to demonstrate the applicability of *WatFinder* to such large systems and to illustrate the computing time for analyzing various size substructures, and in a more detailed analysis focusing on a small number of the subunits.

To make the selections of substructures, the `calcWaterBridgesTrajectory()` function with the default `'chain'` method was used with the options `selstr` and `expand_selection=True` for five starting selections of increasing size. The smallest selection (selection 1) was analyzed initially during methods development and in more detail. **Figure S12** shows a high intensity analysis using the first 30 trajectory frames for selections composed of 1, 4, 8 and 16 subunits. Substructures of 2 and 4 subunits were used together with selection 1 for timing the full pipeline in a more tractable way for **Figure S13**, but not investigated further. **Figure S14** presents a detailed analysis of selection 1 (1 subunit).

Selection 1 was targeted towards a single chain using the selection string `'segname PROA'`. This resulted in 7,825 protein atoms being initially selected, giving a box of dimensions 64 Å x 61 Å x 97 Å, expanding the selection to 12,844 protein atoms and 34,102 atoms in total from one frame. In order to analyze common features across all frames, the selection was expanded further by combining those atoms captured with this protocol across a number of frames, resulting in **141,719 atoms in total** for the high intensity (30-frame) analysis for **Figure S12** and a similar number for the other analyses. The corresponding substructure included most of the atoms from the two neighboring/adjacent chains in the ring and some atoms from other chains including those at the inter-ring interface, summing to atoms contributed from a total of 9 subunits.

The other selections are as follows:

Selection 2 started with 2 adjacent chains (using selection string `'segname PROA PROC'`), resulting in **232,124 total atoms**

Selection 3 started with four adjacent chains forming half a ring (selection `'segname PROA PROC PROH PROD'`; 31,300 initial protein atoms; in a 115 Å x 61 Å x 97 Å box; 168,671 atoms total for one frame), resulting in **410,896 total atoms**

Selection 4 started with a ring of 8 subunits (`'segname PROA PROC PROH PROD PROE PROG PROB PROF'`; 62,600 protein atoms; 175 Å x 168 Å x 101 Å; 275,967 atoms total for one frame), resulting in **550,464 total atoms**

Selection 5 was the full double ring complex (16 subunits; selection `None` or `'all'`), containing 125,200 protein atoms in a box of 197 Å x 188 Å x 207 Å, with **707,044 atoms**.

The high intensity analysis shown in **Figure S12** shows that the time taken by the function `calcWaterBridgesTrajectory()` scales more or less linearly with the final size of the system (R^2 = 0.91). The y-intercept is on the order of two minutes for this case of analyzing 30 frames, which suggests that some steps take this much time regardless of the size of the system.

The full pipeline using 1) `calcWaterBridgesTrajectory` with default `'chain'` method, 2) `savePDBWaterBridgesTrajectory` and 3) `findClusterCenters` was applied to selections starting from 1 chain, 2 chains and 4 chains (see **Figure S13**) using the first 10 frames. We again found a linear relationship for `calcWaterBridgesTrajectory` but with a much smaller intercept, suggesting that the size-independent step is dependent on number of frames.

We believe that this is the step of finding a consistent selection across frames, which is required for downstream steps, as the intercept was close to zero for both cases before adding it.

The second step using `savePDBWaterBridgesTrajectory` also benefited from parallelization by frame. The `findClusterCenters` step, on the other hand, was very fast without parallelization, taking 3 seconds for section 1, 5 seconds for selection 2, and 12 seconds for selection 3, allowing different parameter values to be tested very easily.

We also found larger distances (`distC` parameter) were required to find clusters and these clusters were not easily interpretable, which is likely due to the flexibility of this system. In such a case, *WatFinder* water cluster detection may not give reliable results; therefore, a longer equilibration of the system is recommended, or analysis by trajectory fragments or by a group of structures extracted for example from inspection of the RMSD matrix between pairs of frames or from Principal Component Analysis.

To explore this possibility further, we aligned the trajectory on selection 1 and calculated the RMSD from the initial structure and an RMSD matrix between pairs of frames (**Figure S14a-b**). This confirmed that the beginning of the trajectory was not well equilibrated and differed substantially from the rest, and that there were two subsequent groups of frames with similar conformations within them. We therefore focused our analysis on the 10 frames in the group from frames 20 to 35, discarding the first 10 ns as equilibration time. We still found that `distC=0.8` was required to obtain reasonable numbers of clusters and that many of these were not found in more than `numC=2` frames. But the clusters localized to expected sites (**Figure S14c**), such as the nucleoside-recognition and catalytic parts of the ATP binding sites (**Figure S14d-e**) in line with high-resolution structures of the closed state where the nucleotide can be resolved (**Figure S14f**). Other clusters localized at the inter-ring interface that undergoes a conformational change upon ring closure.

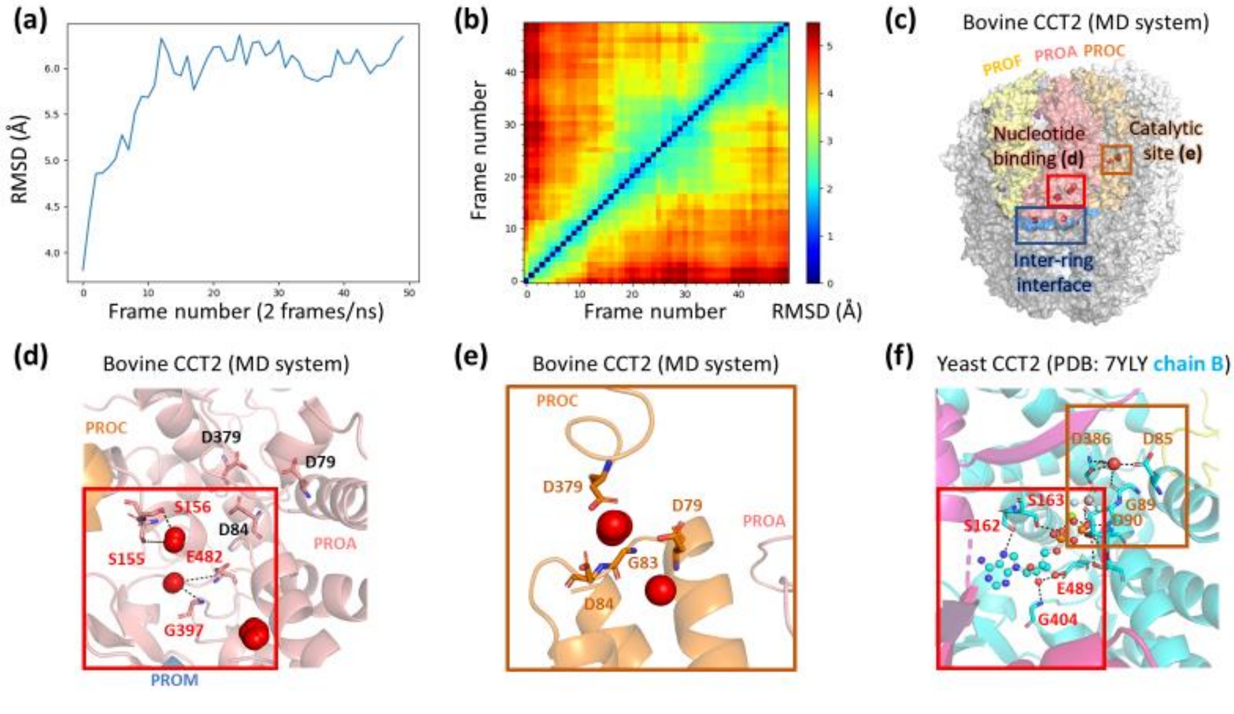

Figure S14. Detailed analysis of selection 1: a single chain and its neighbors. (a) RMSD from the initial structure showing equilibration up to about frame 15 when the RMSD stabilizes at around 6 Å. **(b)** RMSD matrix (color from low RMSD in *blue* to high RMSD in *red*) showing two distinct groups of structurally similar conformers sampled during the simulations, besides the one up to frame 15: one from frame 20 to 35, on which we focused, and another from frame 35 to 50. **(c)** Overview of the selected system shown on frame 22, highlighting water clusters within a distance of 0.8 Å (`distC=0.8`) seen in at least two frames from frame 22 to frame 31 (`numC=2`) as large *red spheres*. The initial target chain (with segment name PROA) is colored in light red and portions of the other chains that are included in the final selection in *yellow* and *orange* for adjacent intra-ring neighbors and *blue* for inter-ring neighbors as in **Figure S12** as well as some smaller portions of other chains in *green* and *purple*. The whole structure is shown with the remainders of the *yellow, orange and blue* colored chains in *gray* and the other chains in *white*. **(d-e)** Zoomed-in views of the water clusters in the nucleotide-binding site, highlighting the ribose, adenine and α-phosphate interacting sites on chain PROA **(d)** and catalytic site on chain PROC **(e)**. **(f)** A comparable view of the nucleotide binding site in a cryo-EM structure (PDB code: 7YLY) (Han et al., 2023), showing the interactions with the hydrolysis transition state analogue ADP-AlF_3 and the catalytic water molecule.

Discussion. This case study shows the application of *WatFinder* to a short MD trajectory (20-25 ns) of a large protein composed of 16 subunits (>8,200 residues) in explicit solvent environment. The *WatFinder* analysis was performed on different numbers of chains to show *WatFinder's* efficiency and scalability.

It was found that the first two steps of analyzing water bridges and saving PDB files could be parallelized across frames, allowing tens of frames to be analyzed simultaneously for significant portions of the trajectory in less than 10 minutes, depending on the number of CPU cores available, which could be helped by using a workstation or cluster node with more cores.

We also found that 10 frames were able to give reasonable results enabling analyses on a laptop or small workstation. It is important, however, to be careful with the alignment of the trajectory using fairly stable substructures, and to analyze the convergence to equilibrium in order to select a well-equilibrated, reasonably conformationally homogenous set of frames to analyze. It may be necessary to be careful with benchmarking analyses on these kinds of large systems as multiple runs sequentially can store multiple results in arrays that overflow the memory.

The final command is very fast and can be easily repeated with different parameters to find the best ones. Our analysis shows that a larger distance and smaller number of frames is more reasonable for such flexible systems.

Conclusion. *WatFinder* enables selection of a subset of a structure and parallel analysis of frames, enabling us to find meaningful clusters at the nucleotide binding site, catalytic site and inter-ring interface within a small number of chains using a subset of frames in the middle of the trajectory, showing the power of *WatFinder* to make sense of trajectories generated for larger systems. The functional importance of these regions may also be an indicator that *WatFinder* could be used to suggest sites for drug or protein design and to investigate protein function. Mutations at the catalytic site are found in some CCT subunits and introducing such a mutation into others has been shown to have drastically different yeast phenotypes (Amit et al. 2010; Kalisman et al. 2013), while inter-ring mutations have critical impacts on allostery in the distantly related GroEL chaperonin from bacteria (Danziger et al., 2003).

WatFinder Parameter Adjustments

WatFinder allows users to choose the method for predicting hydrogen localization as well as various parameters according to the needs of different stages of analysis.

Here is a list of all configurable parameters related to hydrogen bond geometry:

- **distDA** - maximum distance between donor and acceptor atoms in a hydrogen bond that will make a water bridge
- **distWR** - maximum distance between water molecule and protein residue participating in water bridge; waters that do not satisfy this criterion are ignored in further analyses.
- **anglePDWA** - acceptable angle range where the protein is the donor and the water is the acceptor in the formed hydrogen bond
- **anglePAWD** - acceptable angle range when the protein is the acceptor and the water is the donor in the formed hydrogen bond
- **angleWW** - acceptable angle range between two waters forming a hydrogen bond
- **donors** - elements considered as hydrogen bond donors
- **acceptors** - elements considered as hydrogen bond acceptors

All these criteria are used to find hydrogen bonds in a structure (or snapshot from simulations), which are later used to identify possible water bridges. The angles are calculated so that the hydrogen atom that makes a hydrogen bond is the vertex of the angle, and the donor and acceptor atoms are the two ends. This means that the angle is measured between the line connecting the donor to the hydrogen and the line connecting the hydrogen to the acceptor. However, in the absence of data on the position of hydrogen atoms (as is the case for most PDB structures), hydrogen bonds are calculated based on distance and atom types (elements) only.

Here is a list of all configurable water bridge algorithm parameters:

- **maxDepth** - maximum number of water molecules in a chain, or the depth of residues in a cluster
- **maxNumRes** - maximum number of water and protein residues participating in a cluster

These parameters are visualized (in addition to **Fig. 2**) in **Fig. S15**. **Fig. S15** also displays two cases which do not satisfy certain criteria and therefore are not accepted by *WatFinder* as hydrogen bonds or water bridges.

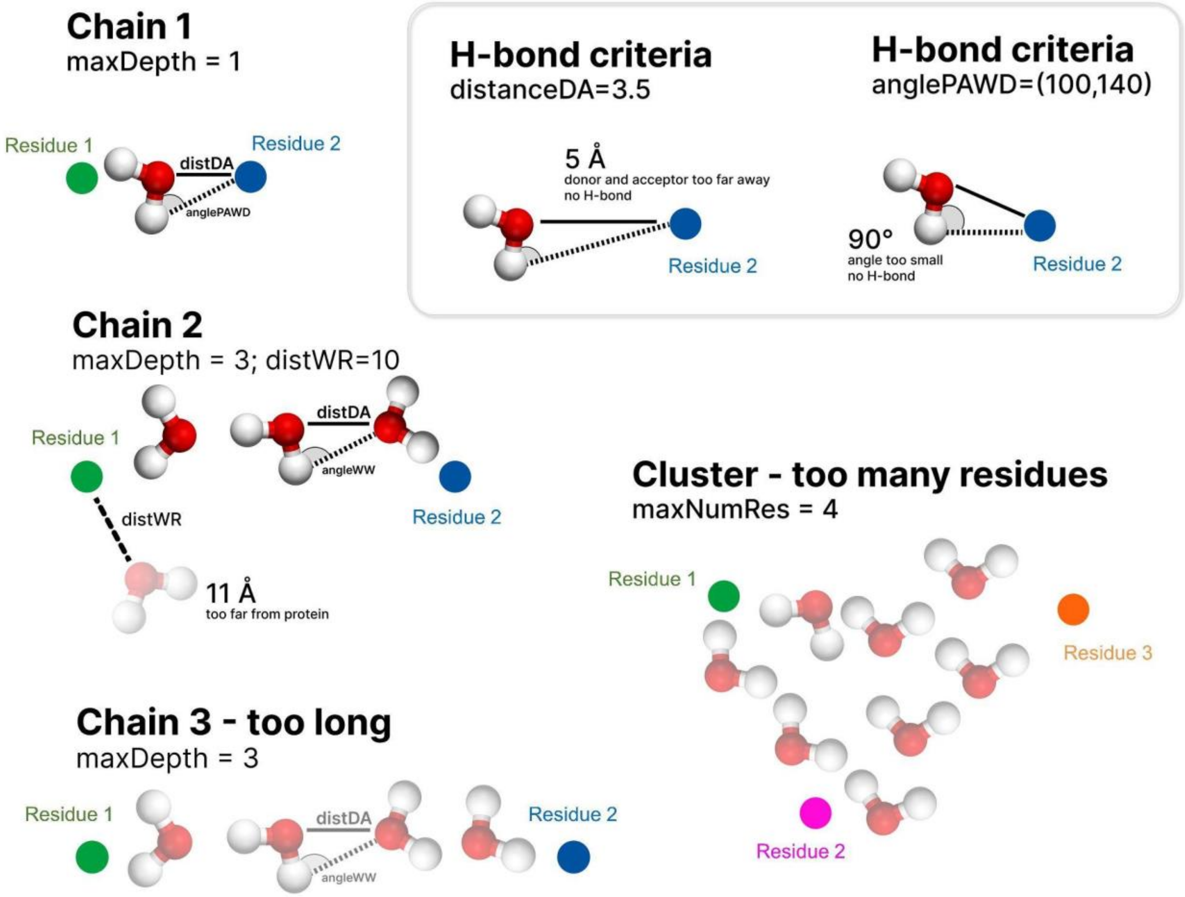

Figure S15. Visual description of parameters used by *WatFinder*.

Here, based on Case Studies 2 (MD trajectory analysis) and 3 (ensemble of crystal structures analysis), we present how different parameters affect *WatFinder's* predictions.

First, we tested what difference it makes if we use *PDBFixer* and *Open Babel* to predict the hydrogen atoms as well as `'chain'` and `'cluster'` methods to predict meaningful protein-water interactions. Case Study 3 evidently implies the crucial role of the method for predicting the position of hydrogen atoms. *Open Babel* seems to insert hydrogens in more favorable places for *WatFinder* predictions. We tested multiple parameters (**Supplementary Table S1**) and each time the *Open Babel* predictions resulted in a larger number of clusters compared to the *PDBFixer's* (*right vs left side of the table*). In Case Study 3, the methods `'cluster'/'chain'` did not show any changes. This was probably due to the analysis of crystallographic waters which were rather not forming water clusters. We can see that changing certain parameters, such as `distDA=4` or `maxDepth=3`, do not change the predictions either. Additionally, we show how the results change if we do not add hydrogen atoms. In such cases, the angular criterion is not considered, and water molecules with unsatisfactory geometry for forming water bridges may be included.

We further tested different parameters, such as `distC` (maximum distance between independent water molecules in the cluster and counted between oxygen atoms in Å) and `numC` (minimum number of water molecules in a cluster) for predicting water clusters using `findClusterCenters()`. These parameters change depending on the tested system and the flexibility of side chains involved in protein-water interactions. The larger `distC`, the more expanded the cluster; and the larger `numC`, the more conserved the detected water cluster. Water clusters W1 and W2, presented in **Fig. S7**, are detected for each prediction for the *Open Babel*, but not all predictions when using *PDBFixer*, due to the angular criteria of hydrogen atoms.

Supplementary Table S1. Summary of different parameters and methods used for *WatFinder* predictions and applied to Aurora kinase A, as shown in Case Study 3. A total number of 156 PDB structures was used.

	**PDBFixer**	**Open Babel**	**No hydrogens**
distC **0.2**<br>*numC* **5**<br>*Method:*<br>**chain**	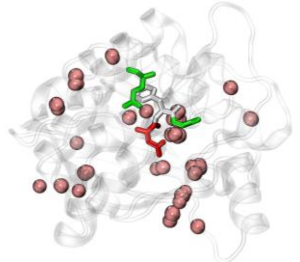	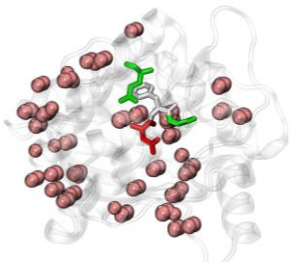	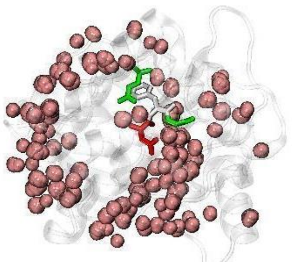
distC **0.2**<br>*numC* **5**<br>*Method:*<br>**cluster**	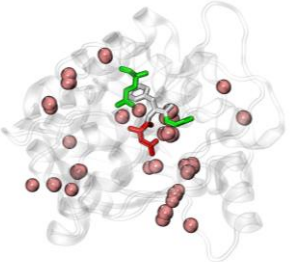	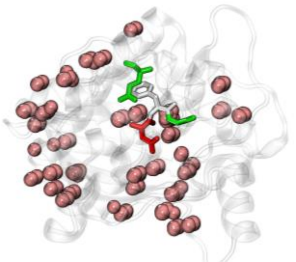	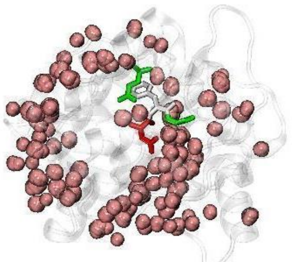
distC **0.35**<br>*numC* **10**<br>*Method:*<br>**chain**	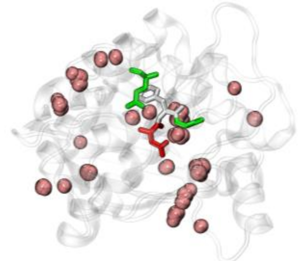	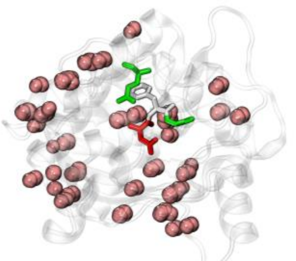	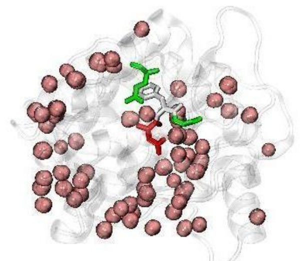
distC **0.35**<br>*numC* **10**<br>*Method:*<br>**cluster**	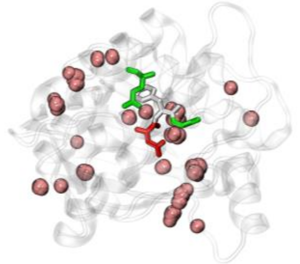	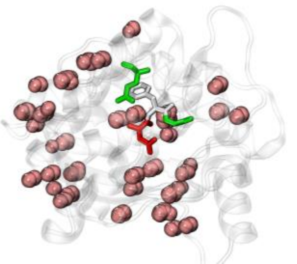	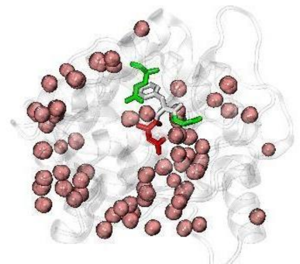

distC **0.35**<br>*numC* **15**<br>*Method:*<br>**chain**	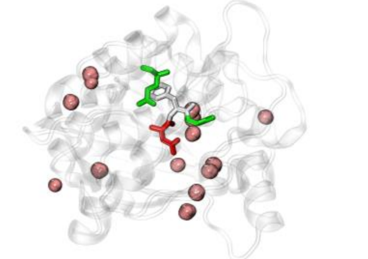	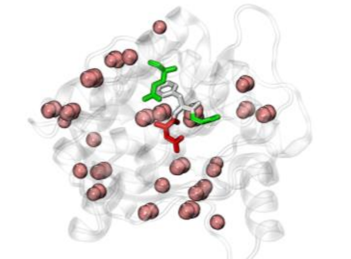	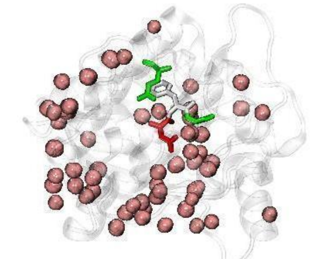
distC **0.35**<br>*numC* **15**<br>*Method:*<br>**cluster**	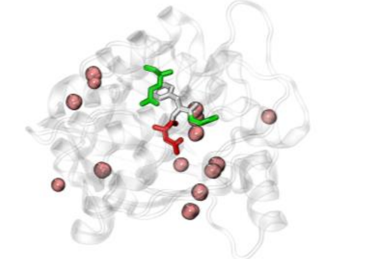	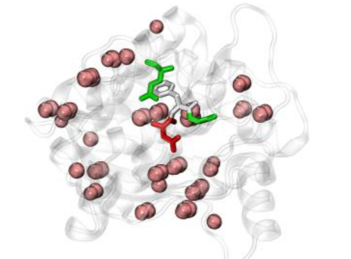	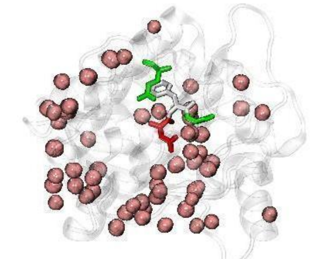
distC **0.4**<br>*numC* **15**<br>*Method:*<br>**chain**	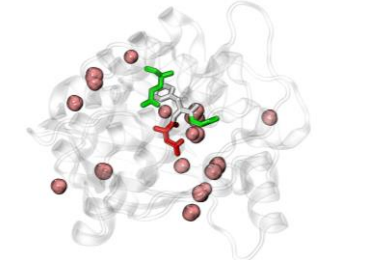	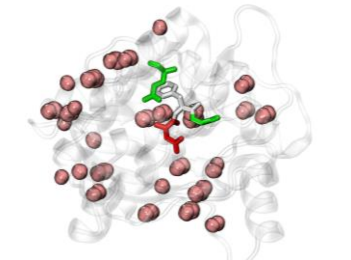	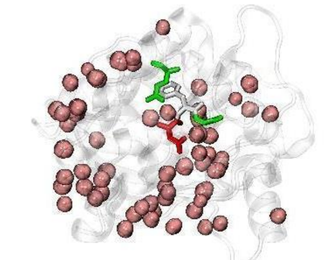
distC **0.4**<br>*numC* **15**<br>*Method:*<br>**cluster**	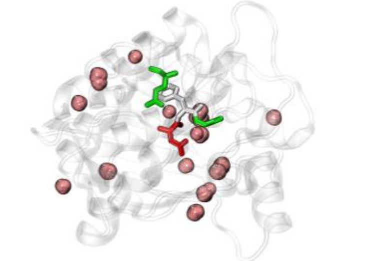	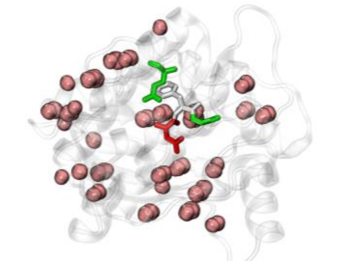	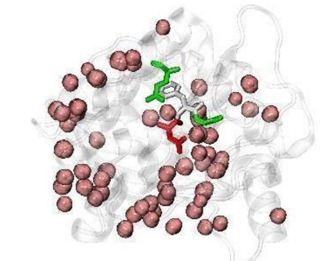
distC **0.5**<br>*numC* **15**<br>*Method:*<br>**chain**	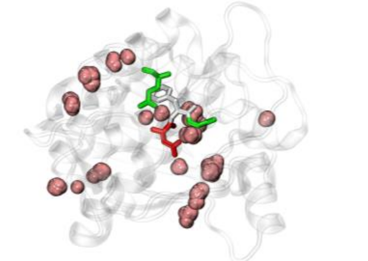	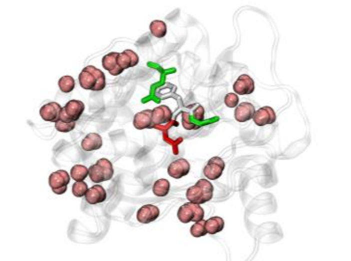	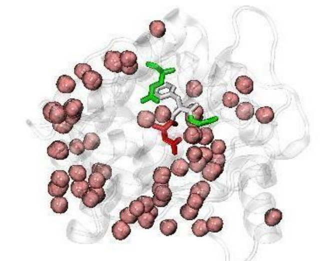
distC **0.5**<br>*numC* **15**<br>*Method:*<br>**cluster**	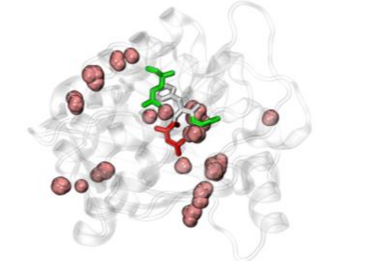	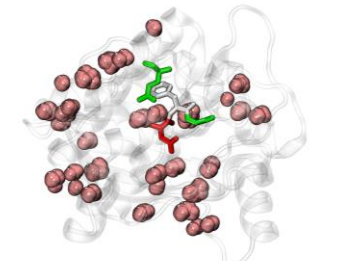	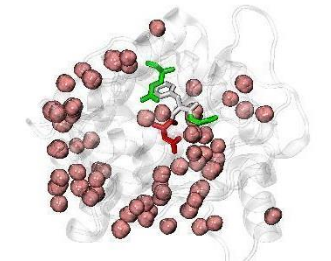

distC **0.5**<br>*numC* **20**<br>*Method:*<br>**chain**	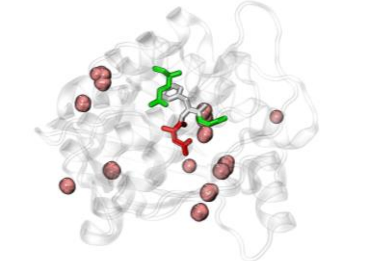	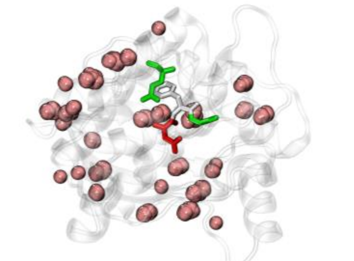	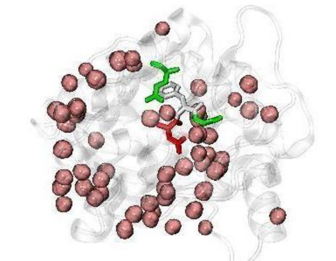
distC **0.5**<br>*numC* **20**<br>*Method:*<br>**cluster**	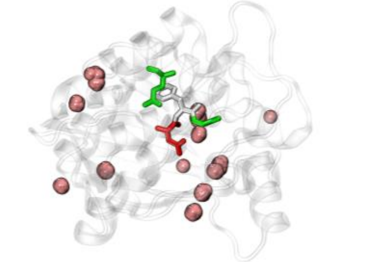	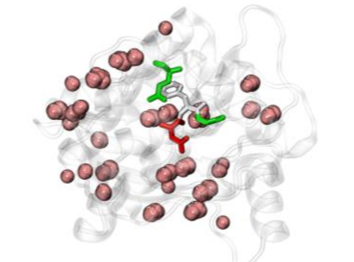	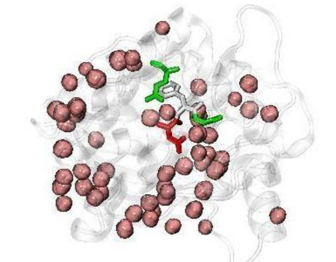
distC **0.7**<br>*numC* **20**<br>*Method:*<br>**chain**	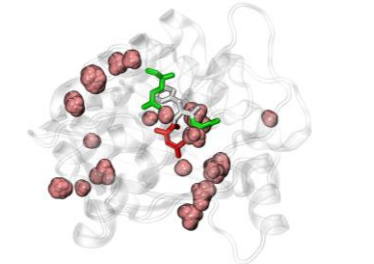	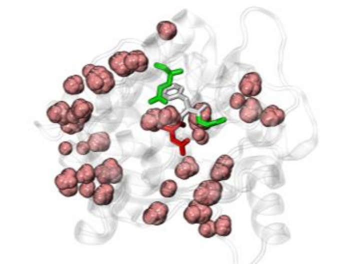	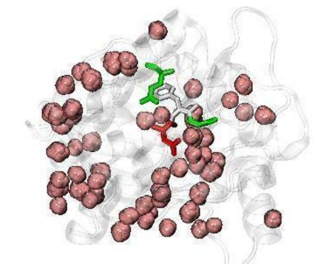
distC **0.7**<br>*numC* **20**<br>*Method:*<br>**cluster**	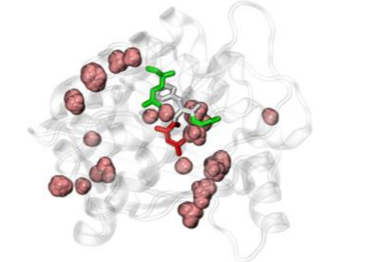	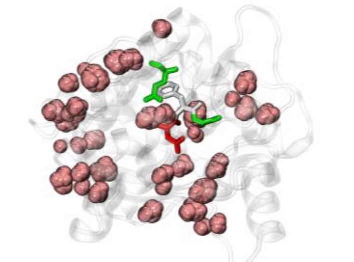	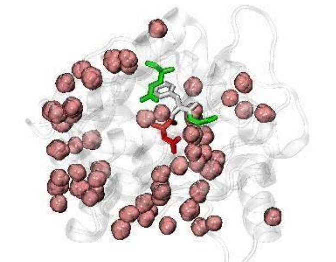

In Case study 3, i.e., the analysis of the MD trajectory, we already had hydrogen atoms. Therefore, we did not test the effect of *Open Babel or PDBFixer* use. Here, we compare the results from the default `'chain'` and the additional and more time-consuming `'cluster'` method that includes multiple residues and waters involved in the protein-water interactions.

In **Supplementary Table S2**, we present the results for VMAT2 trajectory analysis with different parameters comparing the method used to predict protein-water interactions as well as two additional parameters of `calcWaterBridges()` function: `distDA` (distance between donor and acceptor, default 3.5 Å) and `maxDepth` (number of the farthest water included in the calculation, default 2). The results were compared across different `distC` and `numC` parameters applied in `findClusterCenters()` function.

In contrast to the previous case study with crystallographic waters (**Supplementary Table S1**), a high sensitivity to the applied method is observed. The `'cluster'` method includes additional water-water interactions; therefore, it shows slightly better the water flux inside the protein

tunnel. Additionally, by applying higher values of `distDA=4` or `maxDepth=3`, we can increase the number of water molecules in our analysis. In this example, with `distC=0.2` and `numC=10`, we are obtaining only the most conserved protein-water interaction spots. Upon adoption of more relaxed criteria for `distC`, the water flux channel appears. The effect of the relaxed criteria can be observed for `distC=0.5` and `numC=15` or `distC=0.7` and `numC=20`.

Supplementary Table S2. Summary of different parameters and methods used for *WatFinder* predictions and applied to VMAT2 structure, as shown in Case Study 2. Number of frames used for the analysis: 210.

	**Default parameters**	**distDA=4 (chain method) or maxDepth=3 (cluster method)**
distC **0.2**<br>*numC* **10**<br>*Method:*<br>**chain**	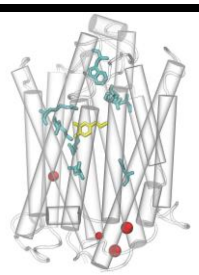	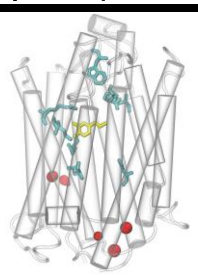
distC **0.2**<br>*numC* **10**<br>*Method:*<br>**cluster**	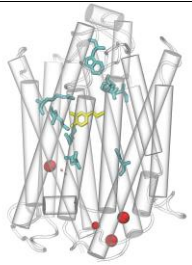	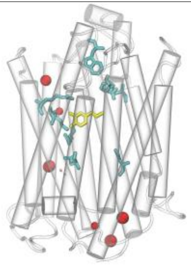
distC **0.3**<br>*numC* **10**<br>*Method:*<br>**chain**	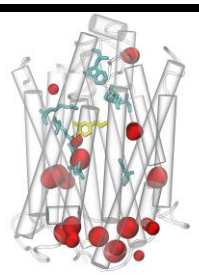	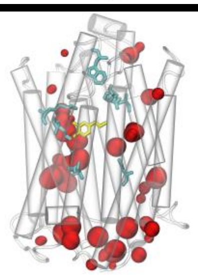
distC **0.3**<br>*numC* **10**<br>*Method:*<br>**cluster**	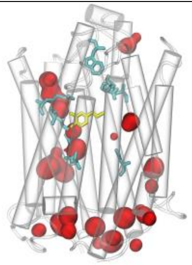	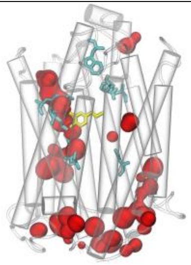

distC **0.35**<br>*numC* **15**<br>*Method:*<br>**chain**	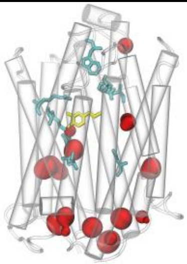	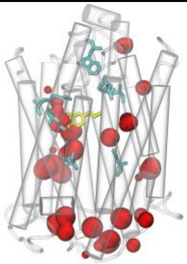
distC **0.35**<br>*numC* **15**<br>*Method:*<br>**cluster**	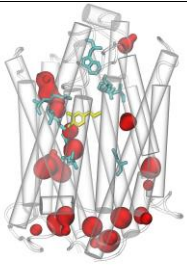	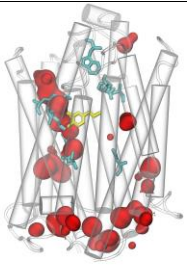
distC **0.4**<br>*numC* **15**<br>*Method:*<br>**chain**	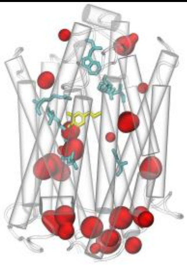	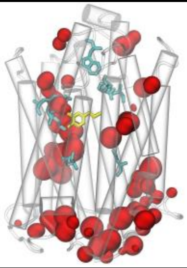
distC **0.4**<br>*numC* **15**<br>*Method:*<br>**cluster**	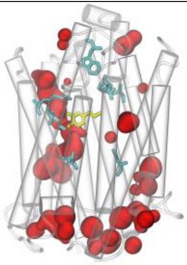	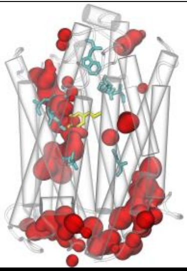
distC **0.5**<br>*numC* **15**<br>*Method:*<br>**chain**	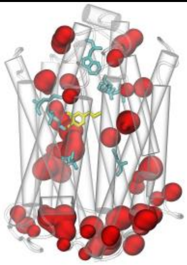	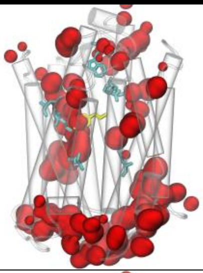
distC **0.5**<br>*numC* **15**<br>*Method:*<br>**cluster**	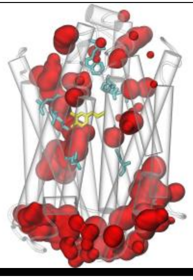	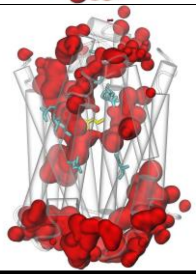

distC **0.5**<br>*numC* **20**<br>*Method:*<br>**chain**	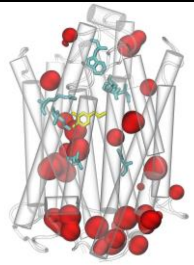	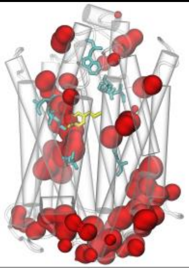
distC **0.5**<br>*numC* **20**<br>*Method:*<br>**cluster**	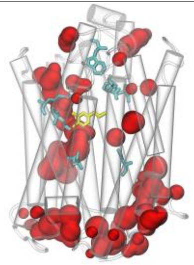	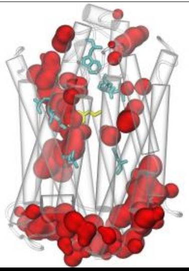
distC **0.7**<br>*numC* **20**<br>*Method:*<br>**chain**	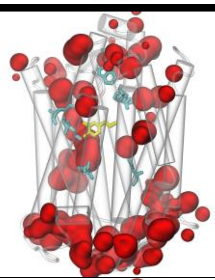	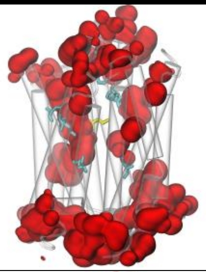
distC **0.7**<br>*numC* **20**<br>*Method:*<br>**cluster**	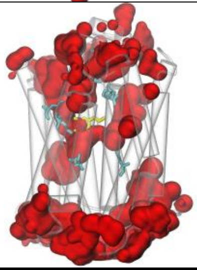	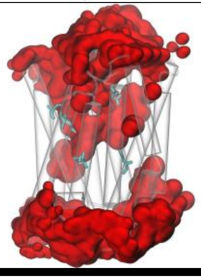

Summary

We presented here the results from a thorough examination of *WatFinder*'s utility, showing various cases and approaches that can be applied to any other system. The systems include examples of an extensive chaperonin system (8,208 residues), a middle-size transmembrane protein, and a small-sized system (214 residues), as well as the usage of ensembles of crystal structures and snapshots from MD trajectories. The cases we used for illustrations provide a wide range of protein types with different functions and environments, including intracellular enzymes and molecular machines, and transmembrane transporters. The studies showed the adaptability and versatility of the *WatFinder* tool and well as the effect of different parameters on the final outcome. Our results show that *WatFinder* may successfully assist in predicting meaningful protein-water interactions in various types of studies, including those confirmed by other studies.

References

Allen, M.P. and Tildesley, D.J. Computer simulation of liquids. Oxford university press; 2017.

Bird, A.W. and Hyman, A.A. Building a spindle of the correct length in human cells requires the interaction between TPX2 and Aurora A. *The Journal of cell biology* 2008;182(2):289-300.

Cyphers, S. *et al.* A water-mediated allosteric network governs activation of Aurora kinase A. *Nature Chem Biol* 2017;13(4):402-408.

Dalton, M.P. *et al.* Structural mechanisms for VMAT2 inhibition by tetrabenazine. *eLife* 2024;12:RP91973.

He, R. *et al.* Inhibition of low molecular weight protein tyrosine phosphatase by an induced-fit mechanism *J Med. Chem.* 2016;59(19):9094-9106.

Humphrey, W., Dalke, A. and Schulten, K. VMD: visual molecular dynamics. *J. Mol. Graph.* 1996;14(1):33-38.

Joung, I.S. and Cheatham III, T.E. Determination of alkali and halide monovalent ion parameters for use in explicitly solvated biomolecular simulations. *J Phys Chem B* 2008;112(30):9020-9041.

Katayama, H. *et al.* Phosphorylation by aurora kinase A induces Mdm2-mediated destabilization and inhibition of p53. *Nature genetics* 2004;36(1):55-62.

Macůrek, L. *et al.* Polo-like kinase-1 is activated by aurora A to promote checkpoint recovery. *Nature* 2008;455(7209):119-123.

Maier, J.A. *et al.* ff14SB: improving the accuracy of protein side chain and backbone parameters from ff99SB. *J. Chem. Theory Comp.* 2015;11(8):3696-3713.

Marumoto, T. *et al.* Roles of aurora-A kinase in mitotic entry and G2 checkpoint in mammalian cells. *Genes to Cells* 2002;7(11):1173-1182.

Pearlman, D.A. *et al.* AMBER, a package of computer programs for applying molecular mechanics, normal mode analysis, molecular dynamics and free energy calculations to simulate the structural and energetic properties of molecules. *Comp. Phys. Commun.* 1995;91(1):1-41.

Phillips, J. *et al.* Scalable molecular dynamics with NAMD. *J. Comput. Chem.* 2005;26(16):1781-1802.

Roe, D.R. and Cheatham III, T.E. PTRAJ and CPPTRAJ: software for processing and analysis of molecular dynamics trajectory data. *Journal of chemical theory and computation* 2013;9(7):3084-3095.

Scheerer, D. *et al.* Allosteric communication between ligand binding domains modulates substrate inhibition in adenylate kinase. *Proc. Natl. Acad. Sci. (USA)* 2023;120(18):e2219855120.

Zhang, S. *et al.* ProDy 2.0: Increased Scale and Scope after 10 Years of Protein Dynamics Modelling with Python. *Bioinformatics* 2021.

Zoete, V. *et al.* SwissParam: a fast force field generation tool for small organic molecules. *J. Comp. Chem.* 2011;32(11):2359-2368.

Cong, Y. *et al.* Symmetry-free cryo-EM structures of the chaperonin TRiC along its ATPase-driven conformational cycle. *The EMBO J.* 2012;31(3):720-730.

Kim, H., Park, J. and Roh, S.-H. The structural basis of eukaryotic chaperonin TRiC/CCT: action and folding. *Molecules and Cells* 2024:100012.

Vanommeslaeghe, K. and MacKerell Jr, A. CHARMM additive and polarizable force fields for biophysics and computer-aided drug design. *Biochimica et Biophysica Acta (BBA)-General Subjects* 2015;1850(5):861-871.

Zhang, Y. *et al.* State-dependent sequential allostery exhibited by chaperonin TRiC/CCT revealed by network analysis of Cryo-EM maps. *Prog Biophys Mol Biol* 2021;160:104-120.
